# Injectable contraceptive continuation and user experiences in Punjab, Pakistan: a non-randomized prospective cohort study protocol

**DOI:** 10.1186/s12905-025-03969-9

**Published:** 2025-09-07

**Authors:** Hannah Tappis, Kanwal Qayyum, Emily Bryce, Aisha Fatima, Megan Christofield, Huma Haider, Afshan Ameen, Syed Muhammad Akbar Gardezi, Fazal Rafay, Fauzia Assad

**Affiliations:** 1 Jhpiego, Baltimore, Maryland USA; 2 Jhpiego, Islamabad, Pakistan

**Keywords:** Family planning, Contraceptive, Injectable contraceptive, DMPA-SC, DMPA-IM, Pakistan, Self-care, Self-injection

## Abstract

**Background:**

Evidence from multiple pilots and post-introduction scale-up initiatives have demonstrated that self-administered subcutaneous depot-medroxyprogesterone acetate (DMPA-SC) has potential to improve contraceptive continuation rates and expand contraceptive access to populations with limited utilization of facility-based health services. Only a few of these studies have been conducted in South Asian countries, and none where most contraceptive use is of non-hormonal methods that require limited to no contact with the health system, leaving policymakers in countries like Pakistan with limited context-specific evidence to guide decisions on whether, how, and for whom to introduce DMPA-SC.

**Methods:**

A prospective cohort study will be conducted in 41 health facilities and surrounding communities in Punjab, Pakistan. The primary objective of the study is to compare the 12-month contraceptive continuation rate for women who receive DMPA-SC with that for women who receive intramuscular depot-medroxyprogesterone acetate (DMPA-IM). The secondary objectives are to compare characteristics and experiences of participants who opt for DMPA-SC with those of women who opt for DMPA-IM, which must be administered by a health worker. Additionally, a sub-study is planned to assess how well women opting for self-injection of DMPA-SC adhere to standards for commodity storage, injection timing, injection technique and waste disposal.

**Discussion:**

This research offers an opportunity to contribute to global efforts to reduce inequities in access to contraceptive method choices, while generating actionable evidence to inform health sector decision-making in Pakistan. Although study sites are limited to health facilities where a woman’s first self-injection of DMPA-SC is supervised by a nurse, midwife, medical officer, Lady Health Visitor, Family Welfare Worker or Family Welfare Councilor, the research protocol and findings will provide a foundation for future studies testing alternative service provision and self-injection support models.

**Trial registration:**

Registered on clinicaltrials.gov as an observational study (NCT05774626).

**Supplementary Information:**

The online version contains supplementary material available at 10.1186/s12905-025-03969-9.

## Background

Depot medroxyprogesterone acetate (DMPA) is a highly effective family planning method that requires administration every three months for continued contraceptive protection [1]. Recent estimates suggest that injectables are currently used by more than 72 million women worldwide and, while injectables are the most common modern contraceptive method in sub-Saharan Africa, they are less common in other geographic regions [2]. However, science is continuously evolving; continued investments in contraceptive technology have led to emergence of new contraceptive methods, formulations and designs with the aim of improving availability and accessibility of contraceptive choices that meet the needs of women and men around the world [3]. Among these advancements is the introduction of a subcutaneous formulation of DMPA (DMPA-SC), which is therapeutically equivalent and has a similar safety profile as the syringe-administered intramuscular formulation (DMPA-IM) but is administered through the Uniject system with a smaller needle and slightly lower hormonal dose [4].

In recent years, evidence has demonstrated that self-injection of DMPA-SC is safe, feasible, acceptable, can lengthen method continuation, and expands contraceptive accessibility to groups with lower health service utilization rates [5]. For example, a 2019 meta-analysis found higher rates of continuation with self-administration of injectable contraception compared to provider administration, and found no difference in reported pregnancies across arms [6]. World Health Organization (WHO) guidelines for sexual and reproductive health self-care interventions promote scale-up of self-administered DMPA-SC as part of strategies to reduce unmet need for contraception and achieve universal access to sexual and reproductive health [7], and there are numerous governments, foundations and civil society organizations around the world involved in DMPA-SC introduction efforts [8].

As research priorities shift from effectiveness trials and pilot studies to implementation research embedded in introduction and scale-up processes, context is a critical consideration [4, 5, 9]. The majority of studies demonstrating potential for DMPA-SC to improve contraceptive access, continuation and autonomy in low-and-middle income countries were conducted in sub-Saharan Africa where, regionally, injectable contraception already comprises the largest share of the method mix [2]. (Table 1) Few have been conducted in contexts like Pakistan where the contraceptive prevalence rate (CPR) has stagnated, and where the most commonly used contraceptive methods are male condoms (27% of all users), female sterilization (27% of all users), and withdrawal (23% of all users)—all methods that require no or one-time contact with health providers and are non-hormonal, and two of which are coital-dependent and male-controlled. Within this context, DMPA-SC is particularly novel as a hormonal, female-controlled, and multi-month acting method. These differentiating features, alongside a collective interest to understand and meet contraceptive needs of women and couples of Pakistan, make DMPA-SC worthy of exploration [10]. The most recent Pakistan Demographic and Health Survey (2017–2018) further illustrates gaps in meeting individuals’ contraceptive needs; one-third of married women who are not using contraception intend to do so in the future, and 56% of women who discontinue use within 12 months of starting a contraceptive method do so because of reasons other than wanting to become pregnant (e.g. method-related reasons, health concerns, cost, inconvenience, etc.) [10]. The potential for DMPA-SC to improve contraceptive continuation, autonomy and experiences in Pakistan has not been studied extensively. Past studies have assessed Lady Health Worker competence in DMPA-SC initiation [11], client satisfaction [12], and the role of non-governmental organizations in promoting DMPA-SC as a means of self-care [13], but none have examined continuation rates or potential of DMPA-SC to reach new users with interests in delaying or avoiding pregnancy.

**Table 1:** Studies measuring DMPA-SC continuation rates in low-and-middle income countries, 2015–2023*

Author (Year), Title	Country	Study arms	Selected outcomes
Cohort studies			
Hernandez et al. T. (2023). Task-shifting and family planning continuation: contraceptive trajectories of women who received their method at a community-based event in Kinshasa, DRC [14]	DR Congo	• Women receiving a modern contraceptive during community-based FP campaign (including DMPA-SC administered by a nursing student)	• 6-month and 9-month continuation rate • Determinants and reasons for discontinuation
Nai et al. (2022). What Distinguishes Women Who Choose to Self-Inject? A Prospective Cohort Study of Subcutaneous Depot Medroxyprogesterone Acetate Users in Ghana [15]	Ghana	• Self-injected DMPA-SC • DMPA-SC administered by family planning providers	• 6-month and 9-month continuation rate • Determinants and reasons for discontinuation
Sherpa et al. (2021). A prospective cohort study to assess the acceptability of Sayana Press among 18–49-year-old women in Nepal [16]	Nepal	• DMPA-SC administered by family planning providers • DMPA-IM administered by family planning providers	• Client satisfaction • 6-month and 9-month continuation rate
Cover et al. (2019). Continuation of self-injected versus provider-administered contraception in Senegal: a nonrandomized, prospective cohort study [17]	Senegal	• Self-injected DMPA-SC • DMPA-IM administered by family planning providers	• 12-month contraceptive continuation rate • Determinants and reasons for discontinuation
MacLachlan et al. (2018). Continuation of subcutaneous or intramuscular injectable contraception when administered by facility-based and community health workers: findings from a prospective cohort study in Burkina Faso and Uganda [18]	Burkina Faso, Uganda	• DMPA-SC administered by CHW in Uganda and by family planning providers at health facilities in Burkina Faso • DMPA-IM administered by CHW in Uganda and by family planning providers at health facilities in Burkina Faso	• 12-month continuation rate
Cover et al. (2018). Continuation of injectable contraception when self-injected vs. administered by a facility-based health worker: a nonrandomized, prospective cohort study in Uganda [19]	Uganda	• Self-injected DMPA-SC • DMPA-IM administered by nurses at public health facilities	• 12-month continuation rate • Determinants and reasons for discontinuation
Cover et al. (2017). Evaluating the feasibility and acceptability of self-injection of subcutaneous depot medroxyprogesterone acetate (DMPA) in Senegal: a prospective cohort study [20]	Senegal	• Self-injected DMPA-SC	• Self-injection competency at 3-months post-training • Acceptability of DMPA-SC self-injection at 3 months post-training (for those discontinuing at that time) and 6-months (for those continuing) • Home storage and disposal practices
Cover et al. (2017). A prospective cohort study of the feasibility and acceptability of depot medroxyprogesterone acetate administered subcutaneously through self-injection [21]	Uganda	• Self-injected DMPA-SC	• Self-injection competency at 3-months post-training • Timeliness of reinjection
Randomized Control Trials			
Burke et al. (2018). Effect of self-administration versus provider-administered injection of subcutaneous depot medroxyprogesterone acetate on continuation rates in Malawi: a randomised controlled trial [22]	Malawi	• Self-injected DMPA-SC • DMPA-SC administered by family planning providers	• 12-month continuation rate • Determinants and reasons for discontinuation • Adverse events

^*^Articles identified through a PubMed search using strategies outlined in Kennedy et al.’s systematic review and meta-analysis on self-administration of injectable contraception published in 2019 [6]

As the government and other stakeholders in Pakistan seek to expand the reach and range of contraceptive options available to meet the population’s needs, context-specific evidence can help guide strategy development and resource allocation. Further research is needed to understand how and where DMPA-SC self-injection may help address unmet need for contraception in Pakistan, including potential differences in DMPA-SC and DMPA-IM user profiles and factors that will support and hinder continuation. Such insights may be critical to provincial decision-makers considering benefits and costs of expanding service offerings to include DMPA-SC for self-injection, particularly where uptake of injectable contraceptive uptake is low and per-unit procurement costs of DMPA-SC are higher than that of DMPA-IM [23].

## Methods/design

### Study design

This will be a non-randomized prospective cohort study. We calculated the sample size necessary to detect a 10% difference in 12-month continuation rates between DMPA-SC and DMPA-IM users with 80% power at 0.05 level of statistical significance. Assuming equal preference for each injectable method, a 12-month continuation rate of 50% for DMPA-IM users, and a retention rate of 75% follow-up following the 4th injection (the equivalent of 12-months of contraceptive coverage), a total of 500 participants are needed in each intervention group. Thus, we anticipate enrolling a total of 1,000 women (500 DMPA-SC clients and 500 DMPA-IM clients).

### Study setting and site selection

Pakistan’s administrative divisions include four provinces (Balochistan, Kyber Pakhtunkwa, Punjab, Sindh), two autonomous territories (Azad Jammu Kashmir, Gilgit-Baltistan) and a federal capital territory (Islamabad), which are further divided into districts, tehsils (sub-districts), and union councils (villages). Health sector governance was decentralized in 2010–11, with a constitutional amendment placing responsibility for health policy, financing and implementation with provincial authorities [24].

This study will be conducted in Punjab province where the modern contraceptive prevalence rate (mCPR) among married women is 27% [10]. One in six married women in Punjab has an unmet need for family planning, and demand satisfied by modern methods is 50% [10]. Mirroring national trends, the modern contraceptive method mix among married women is dominated by male condom use (28% of contraceptive users) and female sterilization (27%). Injectables contribute 6% of the modern method mix [10].

A total of 41 health facilities were selected as study sites across two districts of Punjab, Kasur and Khanewal. These districts were selected in consultation with the Government of Punjab to represent two of the three administrative regions of Punjab (Central and Southern) and based on the following criteria: mix of urban and rural populations, existing utilization of injectable contraceptives and functional government structures. The government-recommended districts of Kasur and Khanewal were endorsed by the project’s Steering Committee. Health facilities were purposively selected as study sites by study team members, in consultation with local authorities, to reflect variations in location (urban/rural), ownership (Department of Health, Population Welfare Department, private sector), and facility type (district headquarter hospitals, tehsil headquarter hospitals, rural health centers, basic health units, family health clinics, family welfare centers, and private clinics).

### Support for family planning service provision

Study team members will visit each selected health facility to confirm staffing, availability of a family planning service delivery area with space for self-injection training, an appropriate storage space with at least three-month supply of family planning commodities (to mitigate risk of stock-outs during the anticipated enrollment period), and a secure location for storage of study documents. If a selected health facility is unable to demonstrate readiness for study implementation, an alternative of the same type may be identified in collaboration with local authorities. All family planning service providers at selected facilities will receive a three-day training, followed by supportive supervision visits, to ensure that they have the necessary knowledge and skills to counsel clients on a full range of contraceptive methods, screen clients according to the WHO’s Medical Eligibility Criteria (MEC), administer DMPA-IM, DMPA-SC, and effectively coach women to self-inject, calculate their reinjection date, and adopt proper waste management techniques. Community health workers serving populations in facility catchment areas will also receive a one-day training on DMPA-SC, MEC and injection techniques to enable them to support self-injection clients, and, together with male social mobilizers, support community engagement activities to generate awareness about the new family planning method and its availability in study districts. Study team members will conduct routine supportive supervision and monitoring visits to confirm continued readiness for family planning service provision and fidelity to counseling and service provision protocols. In the event that a facilities experiences a stock-out during the enrollment period, recruitment of clients will be paused at that site until commodities are supplied and readiness is again confirmed. Once enrollment targets are met, the study team will continue to monitor family planning service provision at these facilities but will not intervene in the management of commodities supplied by the Department of Health or Population Welfare Department.

### Study population

**Fig. 1:**
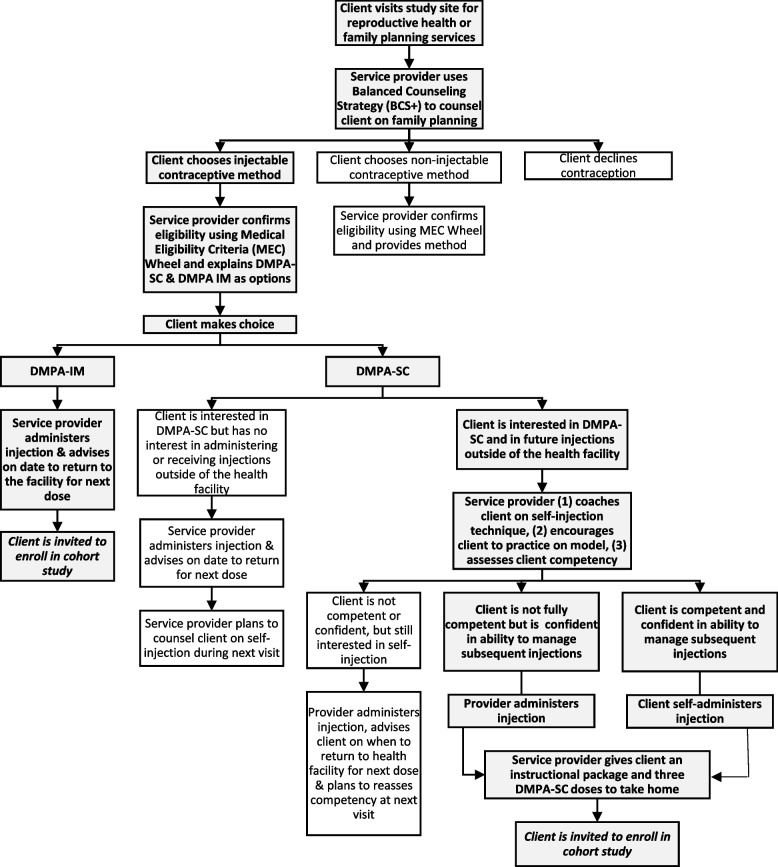
Family planning service provision and cohort study recruitment flow chart

All married women of reproductive age who receive family planning counseling at a participating health facility who choose and are medically eligible to receive injectable contraception will be given an option of either DMPA-IM (current health worker administered standard of care) or DMPA-SC (new administration method, with option for self-injection after completing training). As Fig. 1 shows, those opting for DMPA-SC will receive training on self-injection procedures and an opportunity to practice injection procedures on a model (e.g. condom filled with salt) to demonstrate confidence and competency. The health worker will assess client competency using a standardized checklist; if it is demonstrated, the client will be given the opportunity to inject herself under the supervision of a health worker during the visit. If competency is not demonstrated, or the client does not feel confident enough to self-inject, the health worker will administer the DMPA-SC injection. Both clients that demonstrate competence, and clients who do not demonstrate competence or are lacking confidence to self-inject but remain interested to do so will then be shown how to use a calendar to determine when their next injection is due. Each client will receive a take-home packet that includes an Urdu-language calendar, a pictorial job aid booklet illustrating and the steps for self-injection, safe and appropriate storage, and disposal (Additional file 1), plus three units of DMPA-SC. The job aid booklet also includes a link and QR code that brings users to an animated video demonstrating the same so that they can review self-injection steps at their own time and pace at home. DMPA-SC users will also be advised that they can seek support from community health workers to learn and practice self-injection techniques, and/or seek assistance with administration of their next dose if they still do not wish to self-inject.

#### Inclusion and exclusion criteria

All married women aged 18–49 who receive family planning counseling at a participating health facility and who either receive DMPA-IM or receive DMPA-SC and are given additional doses to take home for future use will be eligible for enrollment in the prospective cohort study. Additionally, to be eligible to participate in the study, women must be residents within the study districts and be willing to be contacted by study team members, either in-person or via telephone.

### Study implementation

This is an ongoing study. All recruitment, enrollment and data collection are expected to take place over an 18-month period.

#### Participant recruitment and enrollment (January – June 2024)

After the client receives their contraceptive method of choice, health workers will provide each client receiving injectables with information about the study, inquire about interest/willingness to learn more about the study, and—if she agrees—obtain informed consent and record her contact information in an enrollment log. Clients providing informed consent to be contacted by study team members will be asked how they would like to be contacted (via telephone or home visit) and contact information recorded. At the end of each workday, health workers will then share enrollment details with study staff, who will contact the client in-person or by phone to confirm eligibility and consent and conduct intake interviews.

Enrollment limits proportional to average family planning client caseload will be set for each facility type, to ensure cohorts include participants from all levels of the health system.

#### Data collection instruments and procedures

Data collection instruments are available in Additional file 2.

### Cohort intake (Tool A) and follow-up (Tool B)

Structured intake interviews will be conducted within two weeks of enrollment. For participants indicating a preference for telephone contact, up to three attempts will be made to make each contact. Participants who indicated a preference for conducting interviews in person, as well as those that could not be reached via telephone and consented to home visits if needed, will be visited at home. Up to three attempts will be made to find a participant in their community. If participants cannot be reached after three attempts via telephone and/or three attempts in person, they will be considered ‘lost to follow up’ and dropped from the study cohort.

Approximately 120–130 days following the date each participant received/administered their contraceptive injection at the health facility and enrolled in the study, participants will be contacted for a follow-up interview (interview #2) using Tool B. This window reflects the expected period of time by which a subsequent injection would have taken place, if conducted within the effective window for DMPA (up to two weeks earlier or four weeks later than the targeted three-month reinjection date), During each follow-up interview, participants will be asked if they received (DMPA-IM) or received/administered another contraceptive injection (DMPA-SC) within the last month. If yes, the study staff will document the date the injection occurred and proceed with the interview on their experiences. If no, the study staff will proceed with questions on discontinuation, and the participant will be dropped from the study cohort.

Approximately 120–130 days following the date each participant reported receiving/administering a second contraceptive injection, they will be contacted for another follow-up interview (interview #3) and the same procedures followed. Similarly, approximately 120–130 days following the date each participant reported receiving/administering a third contraceptive injection, they will be contacted for a final follow-up interview (interview #4) and the same procedures followed. At the end of the interview, participants will be thanked for their participation and informed that the study is completed, and they will not be contacted again.

### Sub-study (Tool C)

During interview #3, DMPA-SC users who live in selected tehsils (sub-districts), are participating in telephone interviews and received/administered a third injection during the study period will be asked if a study staff member can visit their home to better understand how they manage storage, self-injection and waste disposal practices. If the participant agrees, a study staff member will visit their house on an agreed date and time. Upon arrival, the data collector will read a brief consent script to remind participants about the scope and voluntary nature of the study. They will then proceed with questions and visual verification of DMPA-SC product storage conditions, ask participants to demonstrate self-injection procedures using a model like that used in training, and to explain/demonstrate waste disposal practices. DMPA-SC users who live in these tehsils and were not reachable via telephone or indicated a preference for in person follow-up will be visited for interview #3 as described above. At the end of the interview, they will be asked if they are willing to answer a few additional questions and show the study staff how they manage storage, self-injection and waste disposal. If participants agree, study staff will proceed with the interview and observation checklist during the same visit.

#### Criteria for discontinuation of a participant

Consistent with DMPA-SC cohort studies conducted in other low-and-middle income countries, contraceptive discontinuation will be defined as women who did not obtain a scheduled injection, or who self-injected or received any injectable contraceptive more than 120 days after the previous injection. If a participant chooses to discontinue use of contraception or switch to another contraceptive method (either injectable or non-injectable), they will be removed from the study cohort. Participants may also choose to withdraw from participation in the study at any time, with no impact on their access to family planning services.

#### Data management and processing

Data collection will be conducted using Research Electronic Data Capture (REDCap) software on tablet or laptop computers. Routine data management and quality checks will be done in Stata version 14 or higher.

#### Data analysis

Characteristics (e.g. demographics, contraceptive history, reasons for methods choice, reproductive empowerment measured using a validated scale developed by MEASURE Evaluation [25], general self-confidence) and experiences of women enrolled in DMPA-SC and DMPA-IM study arms will be compared using *t* tests for continuous variables and chi^2^ tests for categorical variables. Differences in contraceptive continuation rates of DMPA-IM and DMPA-SC users will be compared at each follow-up point (6,9 and 12-month continuation rates) using Kaplan–Meier survival analysis methods and factors associated with risk of discontinuation analyzed using a Fine-Gray subdistribution hazard model.

Additional descriptive analyses of DMPA-SC user characteristics and experiences may include comparisons of the proportion of clients opting for DMPA-SC who successfully self-inject their first dose under the supervision of a provider; if assistance is sought for subsequent injections; what type of assistance is sought, and from who; and confidence in self-injection capacity.

DMPA-SC client adherence to storage, injection timing, and waste disposal standards will also be measured using descriptive statistics, with sub-analyses comparing differences in adherence based on household demographics, location and access to health services.

### Ethical considerations

Ethical clearance for this study was obtained from the Johns Hopkins Bloomberg School of Public Health (# 21562) and the National Bioethics Committee, Pakistan (# 876).

This is an observational study that will not expose study participants to substantive risks. All participants will provide written informed consent to be contacted by study team members, and oral consent to participate in intake interviews (Tool A), follow-up interviews (Tool B) and, if applicable, home visits (Tool C). Informed consent procedures will be conducted in Urdu, Pakistan’s national language which, along with Punjabi, is the lingua franca in Punjab study districts. All study team members and family planning service providers at participating facilities have been trained in human subjects research ethics, research objectives and consent procedures.

## Discussion

This study will be the first longitudinal study to examine the potential for DMPA-SC to improve access, women’s empowerment, and continuation of family planning in Pakistan. As such, it will help generate evidence to inform Government of Pakistan efforts to expand contraceptive method choice in the context of self-care and support organizations involved in national and provincial efforts to reduce inequities in access to health information, resources and support services.

The study is limited to married women, as it is not socially acceptable to offer contraception to unmarried women or girls in Pakistan. In both public and private sector health facilities, family planning services are only offered to married women [26].

Even so, study findings will add to the growing global evidence base on experiences of DMPA-SC users when given orientation and resources to facilitate self-injection (including home storage, self-injection practices, if assistance is sought from family members/community health workers/others) [27–31] and how continuation compares to DMPA-IM. To the best of our knowledge, only two cohort studies, one conducted in Uganda and the other in Senegal, compared continuation of self-administered DMPA-SC with provider-administered DMPA-IM [17, 19]. Past cohort studies in other low- and middle-income settings have compared continuation of self-administered versus health provider administered DMPA-SC, and of community health worker-administered DMPA-SC with facility-based provider administered DMPA-IM.

According to the most recent Pakistan Demographic and Health Survey, more than half (53%) of all women using family planning each year obtain contraceptives from social marketing outlets, while 26% are served by health facilities (16% by public facilities; 10% by private facilities) and 20% by Lady Health Worker outreach) [10]. Ideally, this study would also include pharmacy and community-based options for first-dose injection, which have shown promise in other settings [32, 33]. However, our study design was constrained by resources available and by provincial policies requiring medical eligibility for injectable contraception be confirmed by a facility-based health service provider (a nurse, midwife, medical officer, Lady Health Visitor, Family Welfare Worker or Family Welfare Councilor). Although other provinces have amended guidelines to allow community health workers to administer the first dose of injectable contraceptives, Punjab province has not [34]. Thus, recruitment sites for this study are limited to purposively selected public and private health facilities providing family planning services. Further research may be needed to test alternative service provision and support models that optimize the potential of DMPA-SC to meet needs of women who want a short-acting contraceptive method that can be obtained and administered with minimal health facility visits.

## Supplementary Information

Additional file 1. Client instructional package (Urdu; English translation created using Microsoft Co-Pilot).

Additional file 2. Data collection tools (English; Urdu version is available upon request).

## Data Availability

Data sharing is not applicable to this article as no datasets were generated or analyzed during the preparation of this study protocol. Deidentified datasets generated through this study will be made publicly available in an open-access repository at doi: 10.6084/m9.figshare.29815553.

## Abbreviations

CPR: Contraceptive prevalence rate
DMPA: Depot-medroxyprogesterone acetate
IM: Intramuscular
mCPR: Modern contraceptive prevalence rate
MEC: Medical eligibility criteria
SC: Subcutaneous
WHO: World Health Organization

## References

[1] Pfizer. Medroxyprogesterone acetate. Sayana press 104 mg/ 0.65 mL Suspension for Subcutaneous (SC) Injection. In. https://labeling.pfizer.com/ShowLabeling.aspx?id=15148.

[2] United Nations Department of Economic and Social Affairs, Population Division. World Family Planning 2022: Meeting the changing needs for family planning: Contraceptive use by age and method. UN DESA/POP/2022/TR/NO. 4. 2022.

[3] Anderson DJ, Johnston DS. A brief history and future prospects of contraception. Science. 2023;380(6641):154–8. 37053322 10.1126/science.adf9341 PMC10615352

[4] Brady M, Drake JK, Namagembe A, Cover J. Self-care provision of contraception: evidence and insights from contraceptive injectable self-administration. Best Pract Res Clin Obstet Gynaecol. 2020;66:95–106. 32199705 10.1016/j.bpobgyn.2020.01.003

[5] Aderoba AK, Steyn PS, Kiarie JN. Implementation strategies to scale up self-administered depot medroxyprogesterone acetate subcutaneous injectable contraception: a scoping review. Syst Rev. 2023;12(1):114. 37403147 10.1186/s13643-023-02216-2 PMC10318699

[6] Kennedy CE, Yeh PT, Gaffield ML, Brady M, Narasimhan M. Self-administration of injectable contraception: a systematic review and meta-analysis. BMJ Glob Health. 2019;4(2):e001350. 31179026 10.1136/bmjgh-2018-001350 PMC6528768

[7] WHO Guidelines Approved by the Guidelines Review Committee. WHO consolidated guideline on self-care interventions for health: sexual and reproductive health and rights. Geneva: World Health Organization; 2019. 31334932

[8] Injectable Access Collaborative. DMPA-SC Advocacy Pack In.: PATH, Clinton Health Access Initiative (CHAI). Supply Health, Jhpiego, JSI; 2023.

[9] Kohn JE. DMPA self-administration can improve contraceptive access, continuation, and autonomy. Lancet Glob Health. 2018;6(5):e481–2. 29526706 10.1016/S2214-109X(18)30077-9

[10] National Institute of Population Studies - NIPS/Pakistan and ICF. Pakistan Demographic and Health Survey, 2017–2018. In. Islamabad, Paksitan and Rockville, Maryland, USA: NIPS and ICF; 2019.

[11] Chin-Quee DS, Abrejo F, Chen M, Lashari T, Olsen P, Habib Z, Gao X, Assad F, Midhet F, Chandio S, et al. Task sharing of injectable contraception services in Pakistan: a randomized controlled trial. Stud Fam Plann. 2021;52(1):23–39. 33742478 10.1111/sifp.12149

[12] Chaudhri R, Rizvi F, Afzal M. Patient satisfaction of depot medroxyprogesterone acetate (DMPA-SC) injection as contraceptive. J Pak Med Assoc. 2010;60(7):536–40. 20578601

[13] Uzma Q, Hamid N, Chaudhri R, Mehmood N, Aabroo A, Thom E, Gholbzouri K, Mahaini R, Hemachandra N. The role of partners in promoting self-care for misoprostol and subcutaneous DMPA in Pakistan. Health Res Policy Syst. 2021;19(Suppl 1):62. 33882966 10.1186/s12961-021-00714-0 PMC8058573

[14] Hernandez JH, LaNasa KH, Koba T. Task-shifting and family planning continuation: contraceptive trajectories of women who received their method at a community-based event in Kinshasa, DRC. Reprod Health. 2023;20(1):24. 36717937 10.1186/s12978-023-01571-6 PMC9887934

[15] Nai D, Tobey E, Fuseini K, Kuma-Aboagye P, Jain A. What Distinguishes Women Who Choose to Self-Inject? A Prospective Cohort Study of Subcutaneous Depot Medroxyprogesterone Acetate Users in Ghana. Global health, science and practice. 2022;10(1):e2100534. 10.9745/GHSP-D-21-00534. 10.9745/GHSP-D-21-00534 PMC8885352 35294390

[16] Sherpa LY, Tinkari BS, Gentle P, Sah RK, Shrestha A, Sahani SK, Aryal K, Ghimire J, Karki DK. A prospective cohort study to assess the acceptability of Sayana Press among 18–49-year-old women in Nepal. Contraception. 2021;104(6):623–7. 34280441 10.1016/j.contraception.2021.07.009

[17] Cover J, Ba M, Drake JK. MD ND: continuation of self-injected versus provider-administered contraception in Senegal: a nonrandomized, prospective cohort study. Contraception. 2019;99(2):137–41. 30439358 10.1016/j.contraception.2018.11.001 PMC6367564

[18] MacLachlan E, Atuyambe LM, Millogo T, Guiella G, Yaro S, Kasasa S, Bukenya J, Nyabigambo A, Mubiru F, Tumusiime J, et al. Continuation of subcutaneous or intramuscular injectable contraception when administered by facility-based and community health workers: findings from a prospective cohort study in Burkina Faso and Uganda. Contraception. 2018;98(5):423–9. 30125558 10.1016/j.contraception.2018.08.007 PMC6197835

[19] Cover J, Namagembe A, Tumusiime J, Nsangi D, Lim J, Nakiganda-Busiku D. Continuation of injectable contraception when self-injected vs. administered by a facility-based health worker: a nonrandomized, prospective cohort study in Uganda. Contraception. 2018;98(5):383–8. 29654751 10.1016/j.contraception.2018.03.032 PMC6197833

[20] Cover J, Ba M, Lim J, Drake JK, Daff BM. Evaluating the feasibility and acceptability of self-injection of subcutaneous depot medroxyprogesterone acetate (DMPA) in Senegal: a prospective cohort study. Contraception. 2017;96(3):203–10. 28673645 10.1016/j.contraception.2017.06.010 PMC6381449

[21] Cover J, Namagembe A, Tumusiime J, Lim J, Drake JK, Mbonye AK. A prospective cohort study of the feasibility and acceptability of depot medroxyprogesterone acetate administered subcutaneously through self-injection. Contraception. 2017;95(3):306–11. 27789309 10.1016/j.contraception.2016.10.007 PMC5356471

[22] Burke HM, Chen M, Buluzi M, Fuchs R, Wevill S, Venkatasubramanian L, Dal Santo L, Ngwira B. Effect of self-administration versus provider-administered injection of subcutaneous depot medroxyprogesterone acetate on continuation rates in Malawi: a randomised controlled trial. Lancet Glob Health. 2018;6(5):e568–78. 29526707 10.1016/S2214-109X(18)30061-5

[23] LaCroix E, Jackson A, McGovern S, Rademacher KH, Rothschild CW. Demand forecasting approaches for new contraceptive technologies: a landscape review and recommendations for alignment. Glob Health Sci Pract. 2023;11(1):e2200334. 36853632 10.9745/GHSP-D-22-00334 PMC9972375

[24] Government of Pakistan Inter-Provincial Coordination Division. Overview of the constitution (Eighteenth Amendment) Act, 2010. 2010.

[25] Chace Dwyer S, Jain A, Ishaku SM, Okunade FT, Uzomba C, Adebayo A, Tobey E. The effect of job aids on knowledge retention among Patent and Proprietary Medicine Vendors trained to administer injectable contraceptives: longitudinal results from implementation science in Nigeria. BMC Public Health. 2019;19(1):1362. 31651273 10.1186/s12889-019-7668-2 PMC6813996

[26] Odwe G, Gray K, Kyarimpa A, Obare F, Nagendi G. Introduction of subcutaneous depot medroxyprogesterone acetate (DMPA-SC) injectable contraception at facility and community levels: pilot results from 4 districts of Uganda. Glob Health Sci Pract. 2018;6(4):711–22. 30429201 10.9745/GHSP-D-18-00117 PMC6370348

[27] Azmat SK. National Policy Landscape Analysis on DMPA-SC, 2023. Islamabad, Pakistan: Jhpiego Pakistan and RIZ Consulting; 2023.

[28] Ruderman LW, Packer C, Zingani A, Moses P, Burke HM. “Men can take part”: examining men’s role in supporting self-injectable contraception in southern Malawi, a qualitative exploration. Reprod Health. 2022;19(1):174. 35945601 10.1186/s12978-022-01476-w PMC9361263

[29] Burke HM, Chen M, Buluzi M, Fuchs R, Wevill S, Venkatasubramanian L, Dal Santo L, Ngwira B. Women’s satisfaction, use, storage and disposal of subcutaneous depot medroxyprogesterone acetate (DMPA-SC) during a randomized trial. Contraception. 2018;98(5):418–22. 29758176 10.1016/j.contraception.2018.04.018

[30] Burke HM, Chen M, Packer C, Fuchs R, Ngwira B. Young women’s experiences with subcutaneous depot medroxyprogesterone acetate: a secondary analysis of a one-year randomized trial in Malawi. J Adolesc Health. 2020;67(5):700–7. 32389457 10.1016/j.jadohealth.2020.03.038

[31] Burke HM, Packer C, Wando L, Wandiembe SP, Muwereza N, Pradhan S, Zingani A, Ngwira B. Adolescent and covert family planning users’ experiences self-injecting contraception in Uganda and Malawi: implications for waste disposal of subcutaneous depot medroxyprogesterone acetate. Reprod Health. 2020;17(1):117. 32746860 10.1186/s12978-020-00964-1 PMC7396890

[32] Burke HM, Thomas R. Thematic analysis and mapping of reproductive empowerment scales: a tool for family planning self-care programming and research. Glob Health Sci Pract. 2022. 10.9745/GHSP-D-21-00794. 36332071 10.9745/GHSP-D-21-00794 PMC9242601

[33] Population Council. Landscape analysis of the family planning situation in Pakistan. Islamabad, Pakistan: Population Council; 2016.

[34] Cover J, Namagembe A, Morozoff C, Tumusiime J, Nsangi D, Drake JK. Contraceptive self-injection through routine service delivery: Experiences of Ugandan women in the public health system. Front Glob Womens Health. 2022;3:911107. 36060608 10.3389/fgwh.2022.911107 PMC9433546

