# Supplementary material for: Injectable contraceptive continuation and user experiences in Punjab, Pakistan: a non-randomized prospective cohort study protocol
Source: BMC Womens Health. 2025 Sep 7;25(Suppl 1):427. doi: 10.1186/s12905-025-03969-9 (PMC12416068; doi:10.1186/s12905-025-03969-9)
Supplement: Supplementary file 1 — Additional file 1. Client instructional package (Urdu; English translation created using Microsoft Co-Pilot). [file 12905_2025_3969_MOESM1_ESM.pdf]

DMPA SC

خود لگانے والا
مانع حمل انجیکشن

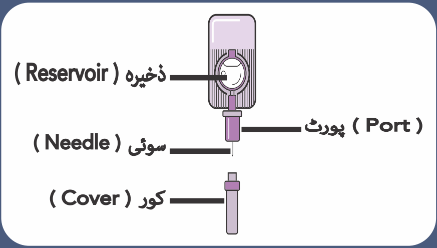

کلائنٹ کے لیے ہدایات

کلائنٹ کا نام ______________________________

ٹیکہ لگانے کی تاریخ __________________________

حکومت پنجاب
متوازن خاندان خوشحال پاکستان
IRMNCH & NUTRITION PROGRAMME
jhpiego
Saving lives. Improving health. Transforming futures.

DMPA-SC ٹیکہ لگانے کے لئے ہدایات

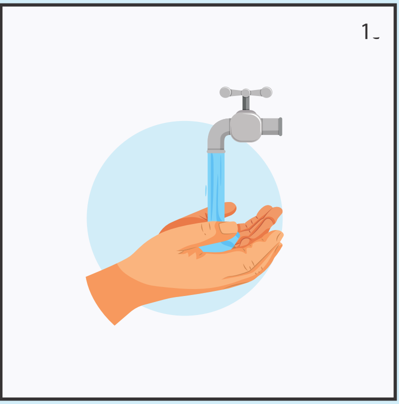

اپنے ہاتھوں کو صاف پانی اور صابن
سے دھوئیں اور خشک کریں۔

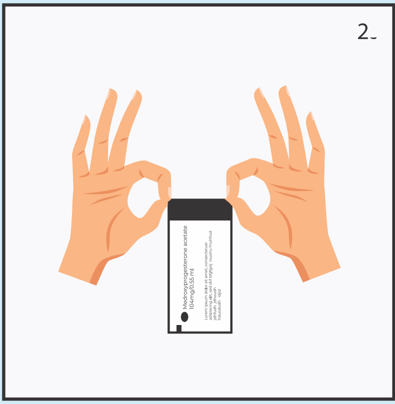

ٹیکے کے پیک پر معیاد ختم
ہونے کی تاریخ (Expiry Date) چیک کریں۔
اگر استعمال کے قابل ہو تو پیکٹ کو کھولیں۔

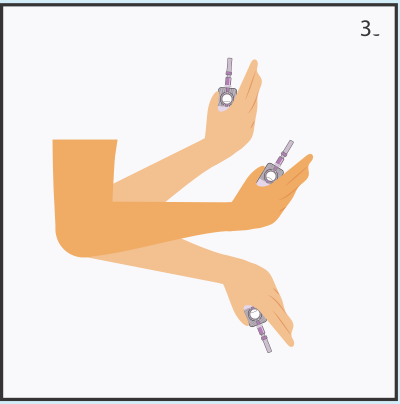

محلول ملانے کے لئے ٹیکے
کو پورٹ کی جگہ سے پکڑ کر
تیس (30) سیکنڈ تک اچھی طرح ہلائیں۔

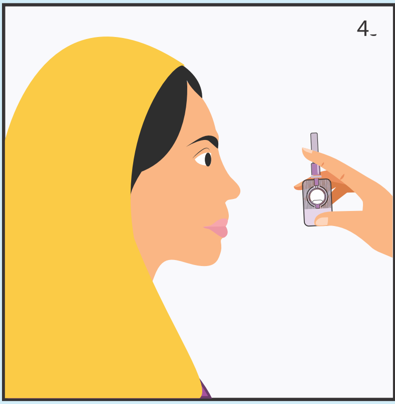

یقین کریں کہ محلول اچھی طرح
مکس ہو گیا ہے اور سوئی ٹوٹی ہوئی
نہ ہو اور نہ لیکیج ہو۔

DMPA-SC ٹیکہ لگانے کے لئے ہدایات

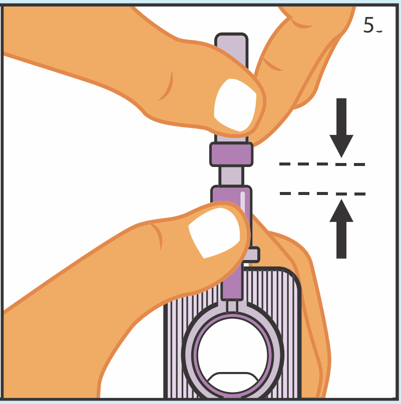

ٹیکے کو پورٹ والی جگہ سے
پکڑیں۔ سوئی کا رخ اوپر کی طرف
ہونا چاہیئے تاکہ ذخیرہ بہنے سے بچے۔

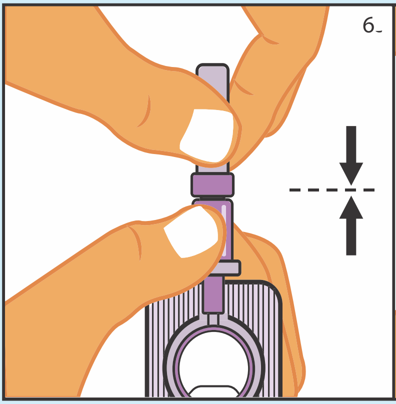

سوئی کے ڈھکنے کو پورٹ تک دبائیں
جب تک سوئی اور پورٹ کا فاصلہ
ختم نہ ہو جائے۔

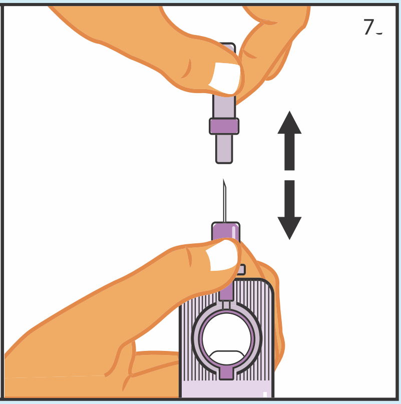

سوئی کے ڈھکن کو ہٹائیں اور استعمال کریں۔

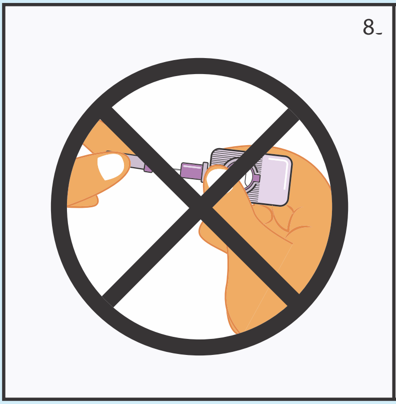

ڈھکن واپس نہ لگائیں۔ ٹیکہ کو استعمال کے
بعد تلف کر دیں۔

DMPA-SC ٹیکہ لگانے کے لئے ہدایات

انجکشن لگانے کے لئے 3 مناسب جگہوں کی نشاندہی

بازو میں، پیچھے کی طرف۔ صحت کارکن یا
آپ کے شوہر آپ کو ٹیکہ لگا سکتے ہیں۔

1۔

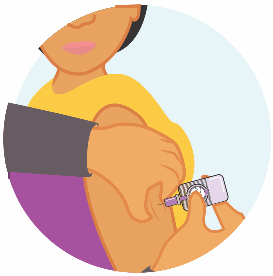

آپ خود پیٹ میں ٹیکہ لگا سکتی ہیں، ناف سے دور

2۔

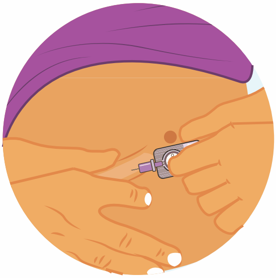

آپ خود ران کے اگلے حصے میں ٹیکہ لگا سکتی ہیں۔

3۔

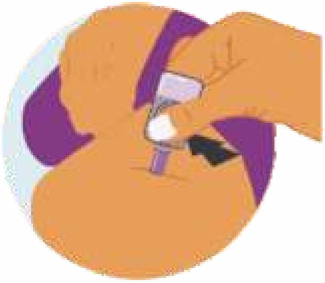

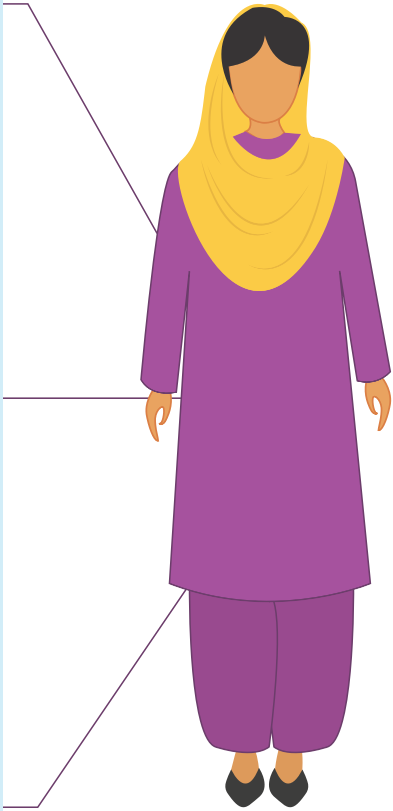

DMPA-SC ٹیکہ لگانے کے لئے ہدایات

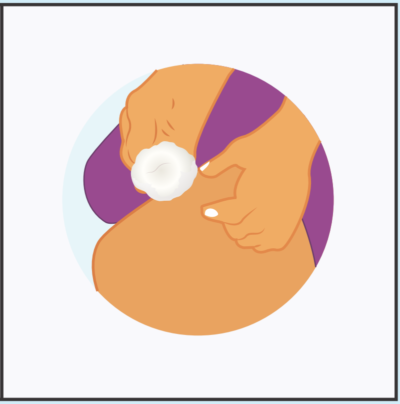

ٹیکہ لگانے والی جگہ کو پانی اور صابن سے صاف کریں۔

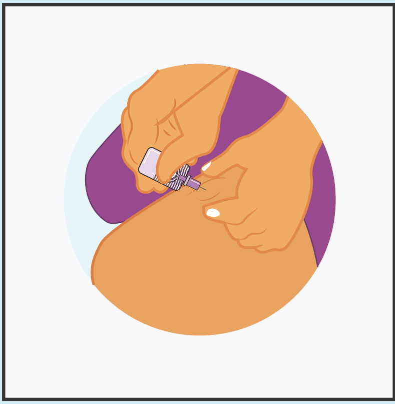

ٹیکہ لگانے کے لیے مناسب جگہ کا انتخاب کریں۔

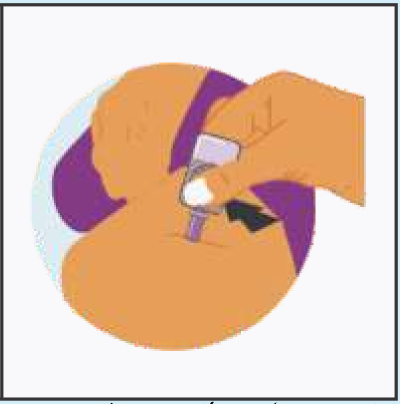

ٹیکے کو پورٹ سے پکڑ کر سوئی کو جلد میں داخل کریں جب تک کہ پورٹ جلد کے ساتھ جُڑ جائے۔ ذخیرے والی جگہ کو 5 سے 7 سیکنڈ تک دبائیں۔ ٹیکہ لگانے کے بعد جگہ کو نہ ہی مسلیں اور نہ ہی صاف کریں۔

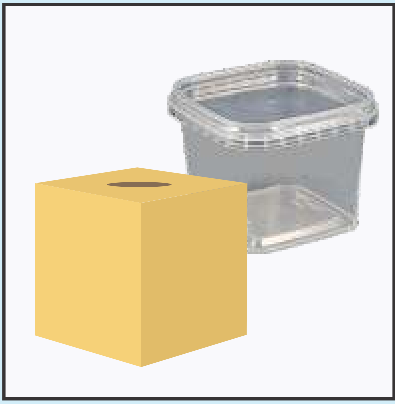

استعمال شدہ ٹیکہ کو تلف کریں۔

DMPA-SC ٹیکہ کو احتیاط سے سنبھالنے کی جگہیں کیا ہو سکتی ہیں؟

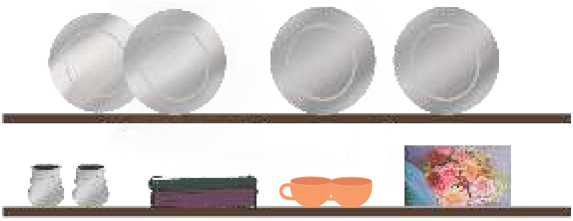

شیلف کے اُوپر بچوں کی پہنچ سے دُور کسی ٹھنڈی اور خشک جگہ پر رکھیں۔

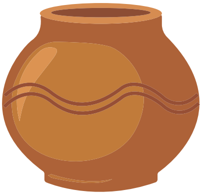

زیادہ درجہ حرارت / گرمی کی صورت میں مٹی کے برتن میں

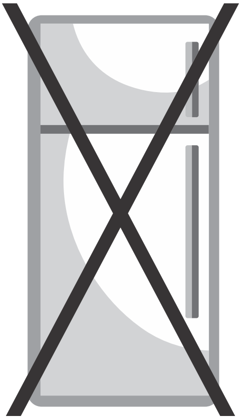

فریج میں نہ رکھیں

DMPA SC خود لگانے والا 3 ماہ کا مانع حمل ٹیکہ

سال میں 4 ٹیکوں کے حساب کا کیلنڈر

یاد سے ٹیکے کو لگانے کے دن کی تاریخ کو نوٹ کر لیں۔ تاکہ 3 ماہ بعد دوبارہ لگا سکیں۔

اگر آپ اپنے اگلے ٹیکے کی تاریخ سے 2 ہفتے پہلے اور 4 ہفتے کے اندر بھی ٹیکہ لگاتی ہیں تو آپ حمل ٹھہرنے سے محفوظ رہیں گی

جنوری 2024

پیر	منگل	بدھ	جمعرات	جمعہ	ہفتہ	اتوار
1	2	3	4	5	6	7
8	9	10	11	12	13	14
15	16	17	18	19	20	21
22	23	24	25	26	27	28
29	30	31				

فروری 2024

پیر	منگل	بدھ	جمعرات	جمعہ	ہفتہ	اتوار
			1	2	3	4
5	6	7	8	9	10	11
12	13	14	15	16	17	18
19	20	21	22	23	24	25
26	27	28	29			

مارچ 2024

پیر	منگل	بدھ	جمعرات	جمعہ	ہفتہ	اتوار
				1	2	3
4	5	6	7	8	9	10
11	12	13	14	15	16	17
18	19	20	21	22	23	24
25	26	27	28	29	30	31

اپریل 2024

پیر	منگل	بدھ	جمعرات	جمعہ	ہفتہ	اتوار
1	2	3	4	5	6	7
8	9	10	11	12	13	14
15	16	17	18	19	20	21
22	23	24	25	26	27	28
29	30					

مئی 2024

پیر	منگل	بدھ	جمعرات	جمعہ	ہفتہ	اتوار
		1	2	3	4	5
6	7	8	9	10	11	12
13	14	15	16	17	18	19
20	21	22	23	24	25	26
27	28	29	30	31		

جون 2024

پیر	منگل	بدھ	جمعرات	جمعہ	ہفتہ	اتوار
					1	2
3	4	5	6	7	8	9
10	11	12	13	14	15	16
17	18	19	20	21	22	23
24	25	26	27	28	29	30

پہلے ٹیکے کی تاریخ ____________ دوسرے ٹیکے کی تاریخ ____________ تیسرے ٹیکے کی تاریخ ____________ چوتھے ٹیکے کی تاریخ ____________

مرکز صحت واپس آنے کی تاریخ ________________________

DMPA SC خود لگانے والا 3 ماہ کا مانع حمل ٹیکہ

سال میں 4 ٹیکوں کے حساب کا کیلنڈر

یاد سے ٹیکے کو لگانے کے دن کی تاریخ کو نوٹ کر لیں۔ تاکہ 3 ماہ بعد دوبارہ لگا سکیں۔

اگر آپ اپنے اگلے ٹیکے کی تاریخ سے 2 ہفتے پہلے اور 4 ہفتے کے اندر بھی ٹیکہ لگاتی ہیں تو آپ حمل ٹھہرنے سے محفوظ رہیں گی

جولائی 2024

پیر	منگل	بدھ	جمعرات	جمعہ	ہفتہ	اتوار
1	2	3	4	5	6	7
8	9	10	11	12	13	14
15	16	17	18	19	20	21
22	23	24	25	26	27	28
29	30	31				

اگست 2024

پیر	منگل	بدھ	جمعرات	جمعہ	ہفتہ	اتوار
			1	2	3	4
5	6	7	8	9	10	11
12	13	14	15	16	17	18
19	20	21	22	23	24	25
26	27	28	29	30	31	

ستمبر 2024

پیر	منگل	بدھ	جمعرات	جمعہ	ہفتہ	اتوار
30						1
2	3	4	5	6	7	8
9	10	11	12	13	14	15
16	17	18	19	20	21	22
23	24	25	26	27	28	29

اکتوبر 2024

پیر	منگل	بدھ	جمعرات	جمعہ	ہفتہ	اتوار
	1	2	3	4	5	6
7	8	9	10	11	12	13
14	15	16	17	18	19	20
21	22	23	24	25	26	27
28	29	30	31			

نومبر 2024

پیر	منگل	بدھ	جمعرات	جمعہ	ہفتہ	اتوار
				1	2	3
4	5	6	7	8	9	10
11	12	13	14	15	16	17
18	19	20	21	22	23	24
25	26	27	28	29	30	

دسمبر 2024

پیر	منگل	بدھ	جمعرات	جمعہ	ہفتہ	اتوار
30	31					1
2	3	4	5	6	7	8
9	10	11	12	13	14	15
16	17	18	19	20	21	22
23	24	25	26	27	28	29

پہلے ٹیکے کی تاریخ ____________ دوسرے ٹیکے کی تاریخ ____________ تیسرے ٹیکے کی تاریخ ____________ چوتھے ٹیکے کی تاریخ ____________

مرکز صحت واپس آنے کی تاریخ ____________________

DMPA SC خود لگانے والا 3 ماہ کا مانع حمل ٹیکہ

سال میں 4 ٹیکوں کے حساب کا کیلنڈر

یاد سے ٹیکے کو لگانے کے دن کی تاریخ کو نوٹ کر لیں۔ تا کہ 3 ماہ بعد دوبارہ لگا سکیں۔

اگر آپ اپنے اگلے ٹیکے کی تاریخ سے 2 ہفتے پہلے اور 4 ہفتے کے اندر بھی ٹیکہ لگاتی ہیں تو آپ حمل ٹھہرنے سے محفوظ رہیں گی

جنوری 2025

پیر	منگل	بدھ	جمعرات	جمعہ	ہفتہ	اتوار
		1	2	3	4	5
6	7	8	9	10	11	12
13	14	15	16	17	18	19
20	21	22	23	24	25	26
27	28	29	30	31		

فروری 2025

پیر	منگل	بدھ	جمعرات	جمعہ	ہفتہ	اتوار
					1	2
3	4	5	6	7	8	9
10	11	12	13	14	15	16
17	18	19	20	21	22	23
24	25	26	27	28		

مارچ 2025

پیر	منگل	بدھ	جمعرات	جمعہ	ہفتہ	اتوار
31					1	2
3	4	5	6	7	8	9
10	11	12	13	14	15	16
17	18	19	20	21	22	23
24	25	26	27	28	29	30

اپریل 2025

پیر	منگل	بدھ	جمعرات	جمعہ	ہفتہ	اتوار
	1	2	3	4	5	6
7	8	9	10	11	12	13
14	15	16	17	18	19	20
21	22	23	24	25	26	27
28	29	30				

مئی 2025

پیر	منگل	بدھ	جمعرات	جمعہ	ہفتہ	اتوار
			1	2	3	4
5	6	7	8	9	10	11
12	13	14	15	16	17	18
19	20	21	22	23	24	25
26	27	28	29	30	31	

جون 2025

پیر	منگل	بدھ	جمعرات	جمعہ	ہفتہ	اتوار
30						1
2	3	4	5	6	7	8
9	10	11	12	13	14	15
16	17	18	19	20	21	22
23	24	25	26	27	28	29

پہلے ٹیکے کی تاریخ ____________ دوسرے ٹیکے کی تاریخ ____________ تیسرے ٹیکے کی تاریخ ____________ چوتھے ٹیکے کی تاریخ ____________

مرکز صحت واپس آنے کی تاریخ ____________________

DMPA SC خود لگانے والا 3 ماہ کا مانع حمل ٹیکہ

سال میں 4 ٹیکوں کے حساب کا کیلنڈر

یاد سے ٹیکے کو لگانے کے دن کی تاریخ کو نوٹ کر لیں۔ تاکہ 3 ماہ بعد دوبارہ لگا سکیں۔

اگر آپ اپنے اگلے ٹیکے کی تاریخ سے 2 ہفتے پہلے اور 4 ہفتے کے اندر بھی ٹیکہ لگاتی ہیں تو آپ حمل ٹھہرنے سے محفوظ رہیں گی

جولائی 2025

اتوار	ہفتہ	جمعہ	جمعرات	بدھ	منگل	پیر
6	5	4	3	2	1	
13	12	11	10	9	8	7
20	19	18	17	16	15	14
27	26	25	24	23	22	21
			31	30	29	28

اگست 2025

اتوار	ہفتہ	جمعہ	جمعرات	بدھ	منگل	پیر
3	2	1				
10	9	8	7	6	5	4
17	16	15	14	13	12	11
24	23	22	21	20	19	18
31	30	29	28	27	26	25

ستمبر 2025

اتوار	ہفتہ	جمعہ	جمعرات	بدھ	منگل	پیر
7	6	5	4	3	2	1
14	13	12	11	10	9	8
21	20	19	18	17	16	15
28	27	26	25	24	23	22
					30	29

اکتوبر 2025

اتوار	ہفتہ	جمعہ	جمعرات	بدھ	منگل	پیر
5	4	3	2	1		
12	11	10	9	8	7	6
19	18	17	16	15	14	13
26	25	24	23	22	21	20
		31	30	29	28	27

نومبر 2025

اتوار	ہفتہ	جمعہ	جمعرات	بدھ	منگل	پیر
2	1					
9	8	7	6	5	4	3
16	15	14	13	12	11	10
23	22	21	20	19	18	17
30	29	28	27	26	25	24

دسمبر 2025

اتوار	ہفتہ	جمعہ	جمعرات	بدھ	منگل	پیر
7	6	5	4	3	2	1
14	13	12	11	10	9	8
21	20	19	18	17	16	15
28	27	26	25	24	23	22
				31	30	29

پہلے ٹیکے کی تاریخ ________ دوسرے ٹیکے کی تاریخ ________ تیسرے ٹیکے کی تاریخ ________ چوتھے ٹیکے کی تاریخ ________

مرکز صحت واپس آنے کی تاریخ ________

ہیلتھ ورکر کے ذریعے DMPA-SC انجیکشن کے لیے مشاہداتی چیک لسٹ

ہیلتھ ورکر کا نام ____________________ تاریخ __________

ہیلتھ ورکر کے مرکزِ صحت کا نام ____________________ ضلع __________

ہر مرحلے کے لیے مطمئن، غیر مطمئن اور نہیں کیا گیا والے خانے میں نشان لگائیں۔

انجیکشن کے مراحل (Steps)	مشاہدہ		
	مطمئن	غیر مطمئن	نہیں کیا
1- چیزیں تیار کریں۔			
2- ہاتھوں کو دھوئیں۔			
3- ٹیکہ لگانے کی جگہ منتخب کریں۔			
4- نشان والی جگہ سے DMPA-SC کا پیکٹ کھولیں۔			
5- DMPA-SC کو صحیح طرح سے مکس کرنے کے لیے اوپر نیچے اچھی طرح 30 سیکنڈ تک ہلائیں۔			
6- ٹیکے کی سوئی کے ڈھکن اور پورٹ کو قابلِ استعمال بنانے کے لیے ایک دوسرے کی طرف دبائیں تاکہ اس کا درمیانی فاصلہ ختم ہو جائے۔			
7- ٹیکے والی جگہ پر چٹکی بھریں۔ اور سوئی کو جلد میں داخل کریں۔			
8- ذخیرے کو کم از کم 7-5 سیکنڈ تک دبائیں تاکہ محلول سوئی کے ذریعے جلد میں داخل ہو جائے۔			
9- ٹیکہ لگانے کے بعد جلد میں چٹکی لگانی والی انگلیوں کو واپس چھوڑ دیں۔			
10- سوئی کے ڈھکن کو لگائے بغیر استعمال شدہ ٹیکے کو فوری طور پر تلف کرنے والے ڈبے میں ڈال دیں۔			

قابلیت کی تشخیص Competency Assessment	ہاں	نہیں
کیا کلائنٹ نے 4 اہم مرحلے (5,6,7,8 مرحلے) مطمئن طریقے سے مکمل کیے؟		

وسائل اور آبادی میں توازن
صحت اور خوشحالی کا ضامن

بچوں کی پیدائش میں مناسب وقفہ
صحت مند ماں اور تندرست بچہ

ماں کا دُودھ متوازن غذا
ماں بچہ تندرست و توانا

وقفہ ہو جب تین سال
ماں صحت مند، گھر خوشحال

Client Instruction Package – DMPA-SC (Translated to English)*

Page 1 (Cover Page)
DMPA-SC Client Instruction Package
Client Name_____
Date of contraceptive injection

Page 2
Instructions for DMPA-SC injection

1. Wash hands with clean water and soap and dry them
2. Check the expiration date. If it is usable, open the package
3. To mix the solution, hold the syringe by the port and shake it well for 30 seconds
4. Make sure the solution is mixed well and the needle is not broken or leaking

Page 3
Instructions for DMPA-SC injection

5. Hold the syringe by the port. The needle should be pointing upwards to prevent the reservoir from leaking.
6. Press the needle cover onto the port until the gap between the needle and the port is gone.
7. Remove the needle cap and use
8. Do not put the cap back on. Discard the injector after use.

Page 4
Instructions for DMAP-SC injection
Identifying three suitable injection sites

- Upper arm
- Abdomen (away from the navel)
- Upper thigh

Page 5
Instructions for DMPA-SC injection

1. Clean the injection site with soap and water.
2. Choose a suitable site for the injection.
3. Press the needle cap and port together to close the gap. Pinch the injection site and insert the needle into the skin. Press the reservoir for 5-7 seconds to allow the solution to enter the skin through the needle. Do not rub or clean the site after injection.
4. Dispose of the used device.

Page 6
What are the possible areas where the DMPA-SC device should be stored?

- Store on a shelf in a cool, dry place out of reach of children.
- In case of high temperature/heat, store in a clay pot.
- Do NOT store inside the refrigerator.

Pages 7-10

Calendar for calculating four injections per year

Remember to note the date so that you can get your next injection after three months have passed. If you get an injection two weeks before it is due, or within 4 weeks after your scheduled injection date, you will be protected from pregnancy.

___Date of 1st injection
___Date of 2nd injection
___Date of 3rd injection
___Date of 4th injection
___Date of return to health center

Page 11

Observation checklist for DMPA-SC injection by a health worker

___Name of health worker
___Name of health facility

Injection steps (Satisfactory/Unsatisfactory/Not done)

1. Prepare injection
2. Wash hands
3. Select injection site
4. Open the DMPA-SC packet from the area marked
5. Shake the device up and down for 30 seconds to mix it properly
6. Press the needle cap and port together to close the gap between them and make the injection usable.
7. Pinch the injection site and insert the needle into the skin.
8. Press the reservoir for 5-7 seconds to allow the solution to enter the skin through the needle.
9. After the injection, release the fingers that pinched the skin.
10. Immediately throw the used device into a disposal container without putting the needle cap on.

Competency assessment: Did the client complete the steps 5-8 satisfactorily? (Yes/No)

*Translation generated using Microsoft 365 Co-Pilot. For verification or additional information about materials used to support DMPA-SC introduction in Punjab, Pakistan, please contact the author team or info@jhpiego.org.
