# Supplementary material for: Injectable contraceptive continuation and user experiences in Punjab, Pakistan: a non-randomized prospective cohort study protocol
Source: BMC Womens Health. 2025 Sep 7;25(Suppl 1):427. doi: 10.1186/s12905-025-03969-9 (PMC12416068; doi:10.1186/s12905-025-03969-9)
Supplement: Supplementary file 2 — Additional file 2. Data collection tools (English; Urdu version is available upon request). [file 12905_2025_3969_MOESM2_ESM.docx]

## **Additional File 2. Study Data Collection Tools**

**Tool A – Enrollment and Interview 1 for DMPA-SC and DMPA-IM clients**

Hello my name is . I work for an organization called Jhpiego. I received your contact number and / address from facility. You agreed that we could contact you for a study about *women’s experiences using injectable contraception.* This study will follow women who chose both DMPA-IM and DMPA-SC to understand their experiences with family planning. The study findings will help the Government of Punjab decide what types of family planning products to make available across the province, and how to best support women and couples to access methods that best meet their needs. If you agree to participate, I will ask you to talk to me about your recent family planning decisions and experiences. During the interview I will make sure that my voice is audible only to you. I will ask you to sit at a quiet place, away from your husband and other family members where you are comfortable to talk and no one can hear our conversation for next 30-60 min. Remember, participation is voluntary and the information you share during our conversation will remain confidential. There is no direct benefit to you from being in this study. You will not be given any financial or non-financial compensation. At any point if you want to take a break, please indicate, we will pause the Interview and resume again. In case our call is disconnected I will call you again. If you have any questions now or during the interview, please ask me and I will try to address your query. I understand that you may find some questions stressful, I will try to help you to manage your stress first. You have a right to leave the study at any point. However, knowing your experience is important to us.

May I start the interview?

Yes

No

| **S/N** | **QUESTION** | **CODES** | **SKIP TO** |
| --- | --- | --- | --- |
| **1. Interview information** | | | |
| 101 | Interviewer Name |  |  |
| 102 | Date of Interview | (DD/MM/YY) |  |
| 103 | Participant ID Number |  |  |
| *104* | *Facility name* |  |  |
| *105* | *Facility location* | Urban  Rural |  |
| *106* | *Facility affiliation authority name* | DoH  PWD  Private |  |
| *107* | *District name* | Kasur  Khanewal |  |
| *108* | *Enrolled in Study Group* | DMPA-SC/Sayana Press  DMPA-IM/Depo |  |
| 109 | Time Interview Started |  |  |
| **2. Eligibility Criteria** | | | |
| 201 | Did you receive an injectable method of contraception during last two weeks? | No  Yes | *If no, stop interview* |
| 202 | Which injectable method did you choose?  *(Probe if unsure)* | DMPA-SC/Sayana Press  DMPA-IM/Depo |  |
| 203 | Did you inject yourself or did a health worker do the injection for you? | Self-injected  Provider injected | *Skip if 202=2* |
| 204 | What is your age? | ____Age in years | *If < 18 years old or > 49 years old, stop the interview* |
| 205 | What is your marital status? | Married  Single  Divorced  Widowed | *If not married, stop the interview* |
| 206 | Do you live in Kasur/ Khanewal district and plan to continue living there for the next year? | No  Yes | *If no, stop interview* |
| 207 | Do you intend to get pregnant in the next 12 months? | No  Yes | *If yes, stop interview* |
| *208* | ***CONFIRMATION***  ***(automated):*** *Is the woman eligible for participation?* | *No*  *Yes* | *If no, stop interview and do not proceed to informed consent* |
| **3. Demographic Information** | | | |
| *Now I want to know some background information about you and your family.* | | | |
| 301 | How many living children do you have? | _____Number of male children (alive)  ____Number of female children (alive) |  |
| 302 | Including you, how many people live in your household?  *(Household = family members who eat from one kitchen).* | ____Number of people |  |
| 303 | Who else lives in your household with you? *(do not read options; select all mentioned)* | Husband/partner  Children  Mother and/or father  Husband’s mother and/or father  Other family members  Lives alone  Other (Specify): |  |
| 304 | What is the highest level of schooling you have attended? | None  Primary School 1-5 grade  Secondary School 6-10 grade  College 11-12 grade  Graduate 13-14 grade  Post graduate and higher |  |
| 305 | What is your occupation? | Unemployed  Housewife  Own business  Professional/business/office job  Sales/retail  Healthcare worker  Teacher  Skilled labor  Unskilled labor  Agriculture  Other (specify) |  |
| 306 | What is the highest level of schooling your husband has attended? | None  Primary School 1-5 grade  Secondary School 6-10 grade  College 11-12 grade  Graduate 13-14 grade  Post graduate and higher |  |
| 307 | What is your husband’s  occupation? | Unemployed  Housewife  Own business  Professional/business/office job  Sales/retail  Healthcare worker  Teacher  Skilled labor  Unskilled labor  Agriculture  Other (specify) |  |
| 308 | Does your household have:   1. Refrigerator 2. Washing machine 3. Sofa 4. Chair 5. Almirah/cabinet 6. Computer 7. Internet Connection   H. Bed | No Yes Don’t know  No Yes Don’t know  No Yes Don’t know  No Yes Don’t know  No Yes Don’t know  No Yes Don’t know  No Yes Don’t know  No Yes Don’t know |  |
| 309 | Does any member of your household have a bank account? | No  Yes |  |
| 310 | What type of fuel does your household mainly use for cooking? | Wood  Natural gas  Other |  |
| 311 | What is the main material of the roof in your household? | Cement / RCC  Other |  |
| 312 | What is the main material of the walls in your household? | Cement / RCC  Other |  |
| 4. **Contraceptive History** | | | |
| *Now, I want know some information related to your contraceptives.* | | | |
| 401 | Before your most recent visit to the facility where you received injectable contraception, have you ever used a family planning method before  to prevent pregnancy? | No  Yes |  |
| 402 | Before this most recent visit where you received injectable contraception, have you used a family planning method to prevent pregnancy in the last  two years? | No  Yes | Skip if 401=No |
| 403 | Which method have you used in the past two years to prevent pregnancy prior to the most recent visit where | Daily pill  IUCD  Injectable  Implant  Male condoms  Female condoms | Skip if 401=No OR if 402=No |
|  | you received injectable contraception?  *(Select all mentioned)* | Emergency contraception  Lactational amen. method  Rhythm/moon beads/safe days  Withdrawal  95. Other (specify): |  |
| 404 | Were you using a family planning method at the time of adopting the  injectable method? | No  Yes | Skip if 401=No OR 402=No |
| 405 | Which method were you using at the time when you switched to the injectable method? *(Select all mentioned)* | Daily pill  IUCD  Injectable  Implant  Male condoms  Female condoms  Emergency contraception  Lactational amen. method  Rhythm/moon beads/safe days  Withdrawal  Other (specify): | Skip if 401=No OR 402= No OR  404=No |
| 406 | Before your most recent facility visit, have you heard that there is a type of injectable that you can inject yourself? | No  Yes |  |
| 407 | From where did you hear about DMPA-SC?    (Select all responses) | Facility-based provider (PWD)  Facility-based provider (DoH)  Lady Health Worker  Family Welfare Assistant  Community-based volunteer  Husband  Mother-in-law  Other family member  Peer  Community session  IEC (leaflet, brochure)  Radio ad  Television ad  Social media (Facebook, etc.)  Other (specify) | If 406=No, skip |
| 408 | Now I have some questions about the future. Would you like to have another child someday, or would you prefer not to have any (more) children? | Have a child / another child  No more children  Undecided / don’t know |  |
| 409 | How long would you like to wait from now before conceiving a/another child? | < 1 year  1-2 years  2-5 years  > 5 years  Don’t know | Skip if 408=2 or 408=3 |
| **5. DMPA IM Participants ONLY** *(If 204=2 or 204=3, skip section 5)* | | | |
| 501 | Why did you choose injectable contraception? | Prevents pregnancy effectively  More effective than previous method  Method is safe  Provider recommended it  Husband recommended it  Friends, relatives recommended it  Available/easy to obtain  Convenient to use  Few side effects  Can easily hide it  Heard about DMPA SC through media  Preferred method was not available  Not eligible for preferred method  Reversible  Long term method  Other (Specify): |  |
| 502 | Did you have any concerns about using injectable contraception? | No  Yes |  |
| 503 | What concerns do you have? | How effective it is at preventing pregnancy  How safe it is to use  Side effects/health problems  Fear of future infertility  Fear of birth defects  Pain during or after injection  Unsure of when to return for re- injection  Unsure if able to return for re- injection  Husband/partner doesn’t approve  Other(specify) | If 502=No, skip |
| 504 | What is the reason or reasons you declined to try self-injection? | Convenient to visit clinic  Coming to clinic anyway  Prefer the clinic  Prefer Depo (IM) to DMPA-SC  Trust the provider to do it  Not discreet to inject at home  Afraid of needles, fear of pain  Worried I will do it wrong  Worried I will give it at the wrong time  Need permission to try it  No time for training  Other (specify) |  |
| 505 | If both methods were administered by a provider, would you prefer to use DMPA IM or DMPA-SC, or do you have no preference? | DMPA-SC/Sayana Press  DMPA-IM/Depo  No preference |  |
| 506 | Why would you prefer this type of injection?    *(Do not read responses. Record all mentioned)* | Fewer side effects  Less painful  Available from LHW/clinic  More widely available, generally  Heard negative rumors about other technique  Recommended by others  More effective  More safe  Needle size  Don't know  95. Other (specify): | If 505=3, skip |
| 507 | In the future, would you consider using a type of injectable that you can inject yourself? | No  Yes  Don’t know |  |
| 508 | Would you consider self-administering an injection at home if an LHW was present to  assist you? | No  Yes  Don’t know |  |
| 509 | Would you consider self-administering an injection at home if a trusted friend or family member knew how to  give you the injection? | No  Yes  Don’t know |  |
| **6. DMPA-SC Participants ONLY** *If 204=4, skip section 6* | | | |
| 601 | Why did you choose injectable contraception?    *(Do not read responses. Record all reasons mentioned)* | Prevents pregnancy effectively  More effective than previous method  Method is safe  Provider recommended it  Husband recommended it  Friends, relatives recommended it  Available/easy to obtain  Convenient to use  Few side effects  Can easily hide it  Heard about DMPA SC through media  Preferred method was not available  Not eligible for preferred method  Reversible  Long term method  Other (specify) |  |
| 602 | Do you have any concerns about using injectable contraception? | No  yes |  |
| 603 | What concerns do you have? | How effective it is at preventing pregnancy  How safe it is to use  Side effects/health problems  Fear of future infertility  Fear of birth defects  Pain during or after injection  Unsure of when to return for re- injection  Unsure if able to return for re- injection  Husband/partner doesn’t approve  Other(specify) | If 602=No, skip |
| 604 | If both methods were administered by a provider, would you prefer to use DMPA IM(Depo) or DMPA-SC,  or do you have no  preference? | DMPA IM (Depo)  DMPA-SC  No preference |  |
| 605 | Why would you prefer this type of injection?  *(Do not read responses. Record all mentioned)* | Fewer side effects  Less painful  Available from LHW/clinic  More widely available, generally  Heard negative rumors about other technique  Recommended by others  More effective  More safe  Needle size  Don't know  95. Other (specify) | If 604=3, skip |
| 606 | Were you able to successfully self-inject at the clinic? | No, was deemed not competent so provider gave injection but plans to self-inject  Yes |  |
| *Now that you have had training and a chance to try self-injection, I’d like to ask a few questions about*  *your experience* | | | |
| 607 | How easy or difficult was it to give yourself the injection?    *(Read answer choices)* | Very difficult to do  Somewhat difficult to do  Somewhat easy to do  Very easy | If 606=No, skip to 610 |
| 608 | What was difficult about it? | Inserting the needle  Expelling the drug/emptying the reservoir  Knowing when to give the injection  Knowing where to give the injection  Following/understanding the booklet instructions  Remembering what to do  Calculating the next injection date  95. Other (Specify):  98. Don’t know / don’t remember | If 607=3 or 4, skip |
| 609 | How confident are you that you gave yourself the injection correctly?  *(Read answer choices)* | Not at all confident  A little confident  Pretty confident  Very confident |  |
| 610 | How well did the training and practice on the model prepare you to give yourself the injection?  *(Read answer choices)* | Not well at all unprepared  A Little  Fairly well  Very well |  |
| 611 | What would make you feel more prepared? | More chances to practice injection  More time with a provider  Using a different model  Other training materials (posters, video, etc)  Other (specify): |  |
| 612 | What level of pain did you feel during the injection?  *(Read answer choices)* | Very painful  A little painful  Nearly painless  Painless |  |
| 613 | What level of pain did you feel after the injection?  *(Read answer choices)* | Very painful  A little painful  Nearly painless  Painless |  |
| 614 | Now that you have received the first injection, how confident are you that you know how to give yourself the injection without the provider with you?  *(Read answer choices)* | Not at all confident  A little confident  Pretty confident  Very confident |  |
| 615 | How confident are you that you can follow the steps in the self- injection booklet without the provider present to guide you?  *(Read answer choices)* | Not at all confident  A little confident  Pretty confident  Very confident |  |
| 616 | How confident are you that you know when to give the injection?  *(Read answer choices)* | Not at all confident  A little confident  Pretty confident  Very confident |  |
| 617 | How confident are you that you can use the calendar you were given to find the date for your next injection?  *(Read answer choices)* | Not at all confident  A little confident  Pretty confident  Very confident |  |
| 618 | How anxious do you feel now at the idea of giving yourself an injection?  *(Read answer choices)* | Very anxious  Pretty anxious  A little anxious  Not at all anxious |  |
| 619 | Why do you feel anxious about giving yourself the injection? | Afraid of pain/fear of needles  Afraid of doing it wrong  Because it is a new thing  95. Other | If 618=4, skip |
| 620 | For your next injection, do you plan to self-inject yourself? | No  Yes |  |
| 621 | For your next injection, do you plan to return to the clinic or seek out a LHW/FWA for help with the injection? | No  Yes, clinic  Yes, LHW  Yes, FWA  Don’t know |  |
| 622 | For your next injection, do you expect that you will likely ask a friend or family member to help you with self-injection or will you manage it yourself? | Will likely ask for help  Will likely do it myself  Don’t know |  |
| 623 | If you ask for help with the injection, who would you ask? | Husband  Friend  Parent or in-law  Other family member  LHW  FWA  Clinic provider  Other (specify):  Don’t know | *If 622=2 or 98, skip* |
| **7. Experience of receiving family planning services at the facility** | | | |
| *Now I would like to ask you about your recent experience at the clinic and your decision to use*  *injectable contraception.* | | | |
| 701 | Was the injectable method your first choice, or were you hoping to use some other method when you  went to the clinic recently? | Injectable method was preferred method  Some other method preferred  Don’t know / don’t remember |  |
| 702 | Which method did you  want? | _____________________ | If 701=1, skip |
| 703 | How long did it take for you to travel from your home to the clinic? | Less than 30 minutes  Between 30 minutes and 1 hour  Between 1 and 2 hours  Between 2 and 3 hours  More than 3 hours  Don’t know |  |
| 704 | How did you arrive at the clinic? | Walked/accompanied with another person  Hired a rikshaw  Public transport/ bus  Own Motorcycle  Taxi  Own car  Other (specify) |  |
| 705 | Did you pay anything for your transportation to get to the clinic? | No  Yes |  |
| 706 | How much did it/Fuel  cost? | Amount paid: | If 705=1, skip |
| 707 | How many other people accompanied you to the  clinic? | Number of other people: |  |
| 708 | Who accompanied you?  *(record all mentioned)* | Husband  Children  Parent  In-law  Other family  Friend  Other (specify) | If 707=0, skip |
| 709 | About how long did you have to wait between when you arrived at the clinic and when a provider met with you? | Less than 30 minutes  Between 30 minutes and 1 hour  Between 1 and 2 hours  Between 2 and 3 hours  More than 3 hours  Don’t know / don’t remember |  |
| 710 | Have you ever visited a  LHW/ FWA to get family planning methods? | No  Yes |  |
| 711 | For family planning services, would you prefer to visit a clinic or see a LHW / FWA in the  community? | Visit the clinic  See a LHW  See a FWA  No preference |  |
| 712 | Why do you prefer visiting this source?  *(Do not read responses. Record all mentioned)* | Easy/convenient  Costs less for method or travel  Don’t have to wait in lines  Visiting for other reasons anyway  Have confidence in the provider/LHW/FWA  Know and like the provider/LHW/FWA  More discrete, private  Preferred method available  Availability of commodities  Have more time with the provider/LHW/FWA  Referred by trusted source (family or CBW)  Other(specify): | If 711=3, skip |
| *I’d also like to ask a few questions about family planning services that you received at the clinic a couple weeks ago. I WILL NOT SHARE YOUR RESPONSES WITH ANYONE* | | | |
| 713 | At the visit with the provider a few weeks ago when you started using injectables, were you given information about the other family planning methods  available? | No  Yes |  |
| 714 | Could you please tell me the family planning methods that the provider gave you information about?    **Do not read choices, record all mentioned** | Daily pill  IUCD  Injectable  Implant  Male condoms  Female condoms  Emergency contraception  Lactational amen. method  Rhythm/moon beads/safe days  Withdrawal | If 713= No , skip |
| 715 | Did the provider give you information about possible side effects or problems you might  have with injectables? | No  Yes |  |
| 716 | Could you please tell me the possible side effects the provider told you about injectables?    *(Do not read choices, record all mentioned)* | Irregular bleeding/spotting  Lack of period  Heavy and/or frequent bleeding  Weight gain  Weight loss  Backaches  Headaches  Abdominal pain  Nausea/vomiting  Decreased libido  Vaginal dryness  Pain at injection site  Mood changes  Weakness or fatigue  Breast tenderness  Acne  Other (specify): | If 715=No, skip |
| 717 | Were you told what to do if you experience any side effects or  problems? | No  Yes |  |
| 718 | Were you told about the possibility of switching to another method if the method you selected was not suitable? | No  Yes |  |
| 719 | At the visit with the provider a few weeks ago, did the provider exchange information with you about which FP methods would be most appropriate for you? | No  Yes  Don’t know / don’t remember |  |
| 720 | At the visit with the provider a few weeks ago, would you say the provider was able to address all your questions? | No  Yes  Don’t know / don’t remember |  |
| 721 | At the visit with the provider a few weeks ago, who made the final decision on which family planning method to use? | Woman/self, alone  Provider  Husband/partner, alone  Woman & partner, jointly  Woman & provider, jointly  Woman, Husband & provider, jointly  Other (specify) |  |
| *Please tell me how much you agree or disagree with the following statements. If you say you disagree, I will ask you to tell me if you strongly disagree or just disagree. If you agree, I will ask you to tell me if*  *you strongly agree, or just agree.* | | | |
| 722 | 1. The family planning providers at the facility treat woman with courtesy and respect. 2. The providers make woman like you feel bad when obtaining contraceptives. 3. Family planning providers meet with woman in a private place where you feel comfortable. 4. Family planning providers gave enough information for you to make a decision about family planning. 5. Family planning providers can be counted on to keep information confidential. 6. The family planning provider is available when you want to meet with him/her | \|  \| Strongly  Disagree \| Disagree \| Agree \| Strongly  Agree \| \| --- \| --- \| --- \| --- \| --- \| \| a \| 1 \| 2 \| 3 \| 4 \| \| B \| 1 \| 2 \| 3 \| 4 \| \| C \| 1 \| 2 \| 3 \| 4 \| \| D \| 1 \| 2 \| 3 \| 4 \| \| E \| 1 \| 2 \| 3 \| 4 \| \| f \| 1 \| 2 \| 3 \| 4 \| |  |
| 723 | Overall are you satisfied with the family planning services and care you received from the providers at the clinic?  *(Read answer options)* | Very dissatisfied  Slightly dissatisfied  Slightly satisfied  Very satisfied |  |
| **8. Women’s self-confidence** | | | |
| *Now I am interested to hear about your general self-confidence. I am going to read you two statements.*  *For each, please tell me if you Strongly disagree, Disagree, Agree, or Strongly Agree* | | | |
| 801 | You feel that you are a person of worth, at least on an equal plane with others | Strongly disagree  Disagree  Agree  Strongly agree |  |
| 802 | You feel that you have a number of good qualities | Strongly disagree  Disagree  Agree  Strongly agree |  |
| 803 | You feel that you do not have much to be proud of | Strongly disagree  Disagree  Agree  Strongly agree |  |
| 804 | You feel equal to your peers (e.g. sisters, friends, colleagues, etc) | Strongly disagree  Disagree  Agree  Strongly agree |  |
| **9. Women’s reproductive empowerment & decision-making** | | | |
| *Now I am going to read you a series of statements. For each, please tell me if you Strongly disagree,*  *Disagree, Agree, or Strongly Agree. Please be honest about how you are feeling today.* | | | |
| 901 | You can initiate conversations about using contraception  with your partner | Strongly disagree  Disagree  Agree  Strongly agree |  |
| 902 | You can share your opinions about how many children you want to have with your  partner | Strongly disagree  Disagree  Agree  Strongly agree |  |
| 903 | When having conversations about sex and reproductive health with your partner, he pays attention to what  you have to say | Strongly disagree  Disagree  Agree  Strongly agree |  |
| 904 | You can tell your partner that you don’t feel like having sex without him getting angry, violent or  threatening to leave | Strongly disagree  Disagree  Agree  Strongly agree |  |
| 905 | You can use contraception even if your partner doesn’t want to | Strongly disagree  Disagree  Agree  Strongly agree |  |
| 906 | Who makes the final decision about whether or not you use contraception? | Myself  My husband  My husband and myself, jointly  My husband’s parents  My parents  Another family member  Health care provider  Community-based Healthcare worker  Other (specify)  Don’t know |  |
| *I have a few more statements to read about social support and social norms. Again, for each statement*  *please tell me if you Strongly Disagree, Disagree, Agree or Strongly Agree, based on your current situation.* | | | |
| 907 | If your partner did not want you to use contraception, you have a friend or family member who could help you convince your partner that you should use contraception | Strongly disagree  Disagree  Agree  Strongly agree |  |
| 908 | If your partner did not want you to use contraception, you have people in your community who could help you convince your partner that you should use contraception | Strongly disagree  Disagree  Agree  Strongly agree |  |
| 909 | Friends or family members you are close to decide when they want to use contraception | Strongly disagree  Disagree  Agree  Strongly agree |  |
| 910 | Friends or family members you are close to use contraception even when their partner does not want them to | Strongly disagree  Disagree  Agree  Strongly agree |  |
| 911 | You can initiate conversations and ask questions about contraception with your health care provider | Strongly disagree  Disagree  Agree  Strongly agree |  |
| 912 | When discussing contraception with your health care provider, s/he pays attention to what you have to say | Strongly disagree  Disagree  Agree  Strongly agree |  |
| *Finally, I have a few more statements to read about your experiences using family planning and your partner’s role in previous family planning and pregnancy decisions. Again, for each statement please tell me if you Strongly Disagree, Disagree, Agree or Strongly Agree, based on your current situation or previous experience.* | | | |
| 913 | My partner has stopped me from using a method to prevent pregnancy when I wanted to use one | Strongly disagree  Disagree  Agree  Strongly agree |  |
| 914 | My partner has made it difficult to use a method to prevent pregnancy when I wanted to use one | Strongly disagree  Disagree  Agree  Strongly agree |  |
| 915 | My partner has made me use a method to prevent pregnancy when I did not want to  use one | Strongly disagree  Disagree  Agree  Strongly agree |  |
| 916 | If I wanted to use a method to prevent pregnancy, my partner would stop me | Strongly disagree  Disagree  Agree  Strongly agree |  |
| 917 | My partner has pressured me to become pregnant | Strongly disagree  Disagree  Agree  Strongly agree |  |
| **Those are all the questions I have for you today. Thank you for your time. If you have any problems or concerns about the injection you receive today, contact the clinic. Reminder that a study team member will follow-up with you after your next injection using the preferred method (telephone or**  **home visit).** | | | |
| *1001* | *Record time (end of*  *interview)* | *Time:* |  |
| *1002* | *Calculate next date of visit after 120 days of the 1^st^ injection (auto calculate)* | *Date:* |  |

**Tool B – Follow-Up Interview for DMPA-SC and DMPA-IM clients**

Hello I am__________ from Jhpiego. I am contacting you again because you agreed to share your

experience using injectable contraception*.* Information that you share here will remain confidential. This information will be treated as anonymous for analysis purposes. During the interview I will make sure that my voice is audible only to you. Please sit at a quiet place, away from your husband and other family members, where you are comfortable to talk and no one can hear our conversation for next 30- 45 min. At any point if you want to take a break, please indicate, we will pause the Interview and resume again. In case our call is disconnected I will call you again. If you have any questions now or during the interview, please ask me and I will try to address your query. I understand that you may find some questions stressful, I will try to help you to manage your stress first. You have a right to leave the study at any point. However, knowing your experience is important to us.

May I start the interview?

Yes

No

| **S/N** | **QUESTION** | **CODES** | **SKIP TO** | |
| --- | --- | --- | --- | --- |
| **1. Interview information** | | | | |
| 101 | Interviewer Name |  |  | |
| 102 | Date of Interview | (DD/MM/YY) |  | |
| 103 | Participant ID Number |  |  | |
| *104* | *Facility name* |  |  | |
| *105* | *Facility location* | Urban  Rural |  | |
| *106* | *Facility affiliation authority name* | DoH  PWD  Private |  | |
| *107* | *District name* | Kasur  Khanewal |  | |
| *108* | *Enrolled in Study Group* | DMPA-SC/Sayana Press  DMPA-IM/Depo |  | |
| 109 | Follow-up Interview number | 3-month  6-month  9-month |  | |
| 110 | Time Interview Started |  |  | |
| **1. Experience with chosen method of injectable contraception** | | | | |
| *I would like to start by talking to you about your experience using the injectable since your last interview a few months ago.* | | | | |
| 201 | Since the last interview have you had any problems at the site where the injection was given, like a reaction or skin  irritation? | No  Yes  Don’t know |  | |
| 202 | What type of injection site reaction did you have?  *(Do not read responses. Record all mentioned)* | Tenderness/pain  Bruising/discoloration  Swelling  Dimpling/indentation  Nodule/bump under the skin  Blistering  Oozing of fluid  Itching  Other (specify): | If 201=No, skip | |
| 203 | Did you seek advice or treatment regarding the reaction? | No  Yes |  | |
| 204 | Who did you contact for advice or treatment for the reaction?    *(Do not read responses. Record all mentioned. Probe to clarify, if needed)* | Public clinic staff  Lady health worker (LHW)  FWA  Pharmacy/drug shop  Private clinic staff  Medical consultant  Other (specify) | If 203=No, skip | |
| 205 | Since the last interview, have you experienced any side effects from the injectable contraceptive? | No  Yes |  | |
| 206 | What side effects have you experienced?  *(Do not read responses. Record all mentioned)* | Irregular bleeding/spotting  Lack of period  Heavy and/or frequent bleeding  Weight gain  Weight loss  Backaches  Headaches  Abdominal pain  Nausea/vomiting  Decreased libido  Vaginal dryness  Pain at injection site  Mood changes  Weakness or fatigue  Breast tenderness  Acne  Other (specify) | If 205=No, skip | |
| 207 | Did you seek advice or treatment for side effects? | No  Yes | *If 205=No, skip* | |
| 208 | From whom did you seek advice or treatment for side effects?  *(Do not read responses. Record all mentioned. Probe to clarify, if needed)* | Public clinic staff  Lady health worker  Pharmacy/drug shop  Private clinic staff  Medical consultant  Other (specify) | *If 205=No, skip* | |
| 209 | How tolerable did you feel the side effects were?  *(Read answer options)* | Not tolerable at all  Moderately intolerable  Slightly intolerable  Completely tolerable | *If 205= No, skip* | |
| 210 | Have the side effects gotten better? | No  Yes |  | |
| 211 | Since the last interview, how many times have you visited a hospital, clinic, a LHW, a FWA, or pharmacy for advice or treatment related to your injection or family planning?    *(Enter 0 if she has not sought advice or help.)* | Number of times: |  | |
| 212 | What were the reason or reasons for your visit(s)?    *(Do not read responses. Record all mentioned. Probe to clarify, if needed)* | Side effects/health concern  Injection site reaction  Decided not to use injection  Disposal of used device  Replacement for lost device  Replacement of defective device  Needed help giving myself injection  Questions about injectables  Needed help with reinjection timing  Wanted different method  Other (specify): | *If 211=0, skip to 301* | |
| 3. **DMPA IM Participants ONLY** *If 105=1, Skip* | | | | |
| 301 | Did you receive an injection of Depo (DMPA-IM) since we last spoke 3 months ago? | No  Yes | *If 301=No, skip to* ***Discontinuation Interview (section 5)*** | |
| 302 | What date did you receive the injection? | (DD/MM/YY)… / /    *If doesn’t know, enter 98/98/9898* | *If 301=No, skip* | |
| 303 | **DATA COLLECTOR:**  Was this in the correct window for reinjection?    ***Do not read question,*** *Review autogenerated date from previous survey and compare to date in 302* | No  Yes |  | |
| 304 | Where did you receive this injection? | Public clinic  Lady health worker / health house  Private clinic  Other (specify): |  | |
| 305 | For the most recent injection, was the provider present and available when you arrived for your injection?  *(Read answer options)* | Yes, provider present  No, provider not present so came back later the same day  No, provider not present; came back on a different day  No, provider not present; went to different clinic/provider  No provider not present; other (specify): |  | |
| 306 | Were both the Depo shot and the syringe available when you arrived for your injection? | No  Yes  Don’t know |  | |
| 307 | What did you do?  *(Do not read responses. Record all mentioned)* | Came back another day  Went to another clinic  Bought Depo from pharmacy/drug shop  Bought syringe from pharmacy/drug shop  Other(specify): | *If 306=Yes, skip* | |
| 308 | How easy or difficult was it to remember when it was time for your next injection?  *(Read answer options)* | Very difficult to remember  Somewhat difficult  Somewhat easy  Very easy |  | |
| 309 | What was difficult about  remembering when it was time for the next injection? *(Do not read responses. Record all mentioned)* | A long time had passed  Too busy  Had no reminder  Lost the appointment card  Didn’t receive an appointment card  Other (specify) | *If 308= 3 or 4, skip* | |
| 310 | What strategies did you use, if any, to help you remember your appointment date? | Appointment card  My husband helped me  My child helped me  A friend/other family member helped me  Provider helped me  LHW helped me  FWA helped me  Used a calendar to keep track  Used my phone to keep track  No strategy used  Other (specify) |  | |
| 311 | How difficult or easy was it for you to go to the clinic or health house for the injection?  *(Read answer options)* | Very difficult  Somewhat difficult  Somewhat easy  Very easy |  | |
| 312 | What made going to the clinic or health house for this injection difficult?  *(Do not read responses. Record all mentioned)* | Distance  Cost of travel  Time required  Work to do  Must leave job  Must leave college  Family reason (sickness or visit)  Personal sickness  Keeping visit secret from husband/family  Other (specify): | *If 311= 3 or 4, skip* | |
| 313 | Did you miss work or college to come go to the clinic or health house for your injection? | No  Yes |  | |
| 314 | How many hours of work  or college did you miss? | # hours: |  | |
| 315 | How did you travel to the clinic or health house? *(Do not read responses. Record all mentioned)* | Walked / accompanied by a person  Public transport  Hired a Rikshaw  Own family motorcycle  Taxi/bus  Own car  Other (specify): |  | |
| 316 | Did it cost anything to  travel to the clinic? | No  Yes |  | |
| 317 | How much did it cost for you personally to travel to the clinic?    *(Not including cost for anyone that accompanied*  *her)* | Cost: PKR | *If 316=No, skip* | |
| 318 | How long did it take you to reach the clinic? | Less than 30 minutes  Between 30 minutes and 1 hour  Between 1 and 2 hours  Between 2 and 3 hours  More than 3 hours  Don’t know |  | |
| 319 | How many other people  traveled with you? | Number of people: |  | |
| 320 | Who traveled with you? | Husband  Children  Parent  In-law  Other family  Friend  Other (specify): | *If 319=0, skip* | |
| 321 | Once you arrived, how long did it take you to see the provider? | Less than 30 minutes  Between 30 minutes and 1 hour  Between 1 and 2 hours  Between 2 and 3 hours  More than 3 hours  Don’t know |  | |
| 322 | Do you know when you are due for your next injection? | No  Yes |  | |
| 323 | What is the date of your next injection? | (DD/MM/YY)… / / | *If 322=No, skip* | |
| 324 | **DATA COLLECTOR CHECK:**  Next re-injection date    **Automatically calculated from Q302** | (DD/MM/YY)… / / |  | |
| 325 | How satisfied are you with Depo (DMPA-IM) as a family planning method?  *(Read answer options)* | Very dissatisfied  Somewhat dissatisfied  Somewhat satisfied  Very satisfied |  | |
| 326 | Why are you satisfied?  *(Do not read responses. Record all mentioned)* | Effective against pregnancy  No side effects  Side effects easy to manage  Available/easy to obtain  Facility is close by  It is available free of cost  Timely facilitation  Availability of service provider/LHW/ FWA  Privacy  95. Other (specify) | *If 325= 1 skip* |  |
| 327 | Why are you dissatisfied? | Ineffective against pregnancy  Side effects  Pain during or after injection  Difficult to obtain  Difficult to travel to clinic regularly  Facility is far away  Paid for the services  Services not available when needed  Service provider/LHW / FWA not available  95. Other (specify) | *If 325= 3 or 4, skip* | |
| 328 | Are you considering changing to a different method or stopping using family planning altogether? | No  Yes, thinking about changing methods  Yes, thinking about stopping family planning | *If 325= 3 or 4, skip* | |
| 329 | How likely are you to recommend to a friend that they try Depo (DMPA IM)?  *(Read answer options)* | Very unlikely  Somewhat unlikely  Somewhat likely  Very likely |  | |
| *Before we finish, I would like to read you two statements about personal confidence. For each, please tell me if you Strongly disagree, Disagree, Agree, or Strongly Agree. Please be honest about how you are feeling today.* | | | | |
| 330 | You feel that you are a person of worth, at least on an equal plane with  others | Strongly disagree  Disagree  Agree  Strongly agree |  | |
| 331 | You feel that you have a number of good qualities | Strongly disagree  Disagree  Agree  Strongly agree |  | |
| 332 | You feel that you do not  have much to be proud of | Strongly disagree  Disagree  Agree  Strongly agree |  | |
| 333 | You feel equal to your peers (e.g. sisters, friends, colleagues, etc) | Strongly disagree  Disagree  Agree  Strongly agree |  | |
| ***Those are all the questions I have for you today.***    **IF THIS IS THE 3 OR 6 MONTH FOLLOW-UP, PLEASE READ: *Remember that a study team member will follow-up with you after your next injection using the preferred method (telephone or home visit)***    **IF THIS IS THE 9 MONTH FOLLOW-UP, PLEASE READ: *Thank you for participating in this study. This is our final interview.*** | | | | |
| 4. **DMPA-SC Participants ONLY** If 105=2, skip | | | | |
| *Now I’d like to find out about your most recent experience with self-injection, the one you were due to give yourself 4 weeks ago. Remember that you are always free to change your mind about self-injection, or free to decide to stop using the injectable at all.* | | | | |
| 401 | Did you have an injection of DMPA-SC since the last interview? Either self- injected or injected by  someone else? | No  Yes | *If 401=No, stop and administer* ***Discontinuation interview (section 5)*** | |
| 402 | Did you give yourself the most recent injection? | No  Yes |  | |
| 403 | Who gave you your most recent injection? | Husband  Friend  Family member  Public clinic staff  Private clinic saff  LHW  FWA  Other (specify) | *If 402=Yes, skip* | |
| 404 | What was the date of your last injection?    *If client does not know exact date, look at the auto calculated exact date* | (DD/MM/YY)… / / |  | |
| 403 | **DATA COLLECTOR CHECK:**    Was this in the correct window for reinjection?    ***Do not read question,*** *review calendar/ auto calculated date to confirm if it was in window. If calendar is not available, use enrollment date to calculate window and compare to date in 402* | No  Yes |  | |
| 404 | Were you able to keep the device/injection safe and  secure until you used it? | No  Yes |  | |
| 405 | Please describe what happened, and if there were any consequences  from its discovery | *Open text* | *If 404= Yes, skip* | |
| 406 | Were you able to store it in a cool, dry area at room  temperature | No  Yes  Don’t know |  | |
| 406 | Where did you store the device until you used it?    *(Do not read responses. Record all mentioned)* | Clay pot  Cabinet  Locked box  With other reproductive health supplies  Cupboard  showcase/ with utensils  In side jeweler box  Refrigerator  Other (specify) |  | |
| 407 | How easy or difficult was it to remember when it was time for the injection? | Very difficult  Somewhat difficult  Somewhat easy  Very easy |  | |
| 408 | What was difficult about remembering when to inject? | A long time had passed  Too busy  Had no reminder  Lost the appointment card  Didn’t receive an appointment card  Other (specify) | *If 407=3 or 4, skip* | |
| 409 | What strategies did you use, if any, to help you remember your reinjection date? | Appointment card  My husband helped me  My child helped me  A friend/other family member helped me  Provider helped me  LHW helped me  FWA helped me  Used a calendar to keep track  Used my phone to keep track  No strategy used  Other (specify): |  | |
| 410 | Between your prior injection and this most recent one, did you seek any support for administering self-  injection? | No  Yes |  | |
| 411 | From whom did you seek support for administering self injection? | LHW (DoH)  FWA (PWD)  Facility-based provider  Private clinical provider  Peer  Other, specify |  | |
| 412 | Why did you seek support for administering self- injection? | Practice on administering self- injection  Reminder when to self-inject  Was afraid to do itself  Forgot the technique  Other, specify |  | |
| 413 | Where were you when you gave the injection? | At home  At the LHW’s house  At the FWA’s house  At a friend’s house  Outside  In the latrine  At a clinic  Other (specify): |  | |
| 414 | Where on your body did you give the injection? | Thigh  Abdomen  Other (specify): |  | |
| 415 | Did anyone help you with the injection? | No  Yes |  | |
| 416 | Who helped you with the injection? | Husband  Family member  Friend  Public HF provider  Private Clinic provider  LHW  FWA  Other (specify) | *If 415=No, skip* | |
| 417 | How did they help you? | Helped me read the booklet instructions  Helped me prepare the device  Helped me remember the injection  Injected for me  Observed and guided me  Other (specify) | *If 415=No, skip* | |
| 418 | How easy or difficult was it to give the injection?    *(Read answer options)* | Very difficult  Somewhat difficult  Somewhat easy  Very easy |  | |
| 419 | What was difficult about giving the injection?  *(Do not read responses. Record all mentioned)* | Preparing the unit for injection  Activating the device  Uncapping the device  Inserting the needle  Expelling the drug/emptying the reservoir  Knowing when to give the injection  Knowing where to give the injection  Following the booklet instructions  Remembering what to do  Calculating the next injection date  Don’t know  Other (Specify) | *If 418= 3 or 4, skip* | |
| 420 | What level of pain did you feel during the injection?    *(Read answer options)* | A lot of pain  A little pain  Nearly painless  Painless |  | |
| 421 | What level of pain did you feel after the injection?    *(Read answer options)* | A lot of pain  A little pain  Nearly painless  Painless |  | |
| 422 | Did you use the client instruction booklet to remember the injection steps? | No  Yes |  | |
| 423 | What is the reason or reasons you did not use the booklet? | Didn’t need it to remember how to inject  Lost the booklet  Forgot the booklet  Booklet was too hard to understand  I cannot read  Injected with help from another person  Other (specify) | *If 422= Yes, skip* | |
| 424 | How confident are you that the injection was done correctly?    *(Read answer options)* | Not at all confident  A little confident  Pretty confident  Very confident |  | |
| 424 | What did you do with the device/injection after your administration?    *(Do not read responses. Record all mentioned)* | Threw it in the latrine  Put it in a sealed container  Threw it in the garbage at my house  Threw it in a garbage dump in my area  Burned it  Brought it to the clinic  Brought it to the LHW  Brought it to the FWA  Other (specify) |  | |
| 426 | Did you use the reinjection calendar to calculate your next injection date? | No  Yes |  | |
| 427 | What is the reason you did not use the calendar? | Didn’t need it to calculate the date  Used a different calendar  Lost the calendar  Wasn't given any calendar  Forgot about the calendar  Calendar was too hard to understand  Did not know how to use the calendar  Got help another person  Other (specify) | *If 426=Yes, skip* | |
| 428 | What is the date for your next injection?    *If client does not know her injection date, give her the opportunity to calculate it now, using the booklet and calendar for assistance* | (DD/MM/YY) ___ / / ___ |  | |
| 429 | How confident are you that you calculated the date correctly?    *(Read answer options)* | Not at all confident  A little confident  Pretty confident  Very confident |  | |
| 430 | What concerns do you have about self-injection, if any? | No concerns  Doing the steps correctly  That it is painful  Side effects  Remembering to give the injection  Storing the device  Disposing of waste  Calculating the dates correctly  Someone finding the device  Husband disapproval  Other (specify): |  | |
| 431 | How anxious do you feel at the idea of giving yourself an injection now?    *(Read answer options)* | Very anxious  Somewhat anxious  A little anxious  Not anxious at all |  | |
| 432 | How satisfied are you with DMPA-SC as a family planning method?    *(Read answer options)* | Very dissatisfied  Slightly dissatisfied  Slightly satisfied  Very satisfied |  | |
| 433 | Why are you satisfied with DMPA-SC? | Effective against pregnancy  No side effects  Side effects easy to manage  Available/easy to obtain  Facility is close by  It is available free of cost  Timely facilitation  Availability of service provider/CHW  Its mode of administration | *If 432=1 or 2, skip* | |
| 434 | Why are you not satisfied with DMPA-SC? | Ineffective against pregnancy  Side effects  Pain during or after injection  Difficult to obtain  Difficult to travel to clinic  Facility is far away  Paid for the services  Services not available when needed  Service provider/CHW not available  Its mode of administration | *If 432=3 or 4, skip* | |
| 435 | How likely are you to recommend to a friend using DMPA-SC?    *(read answer options)* | Very unlikely  Somewhat unlikely  Somewhat likely  Very likely |  | |
| 436 | How likely are you to recommend to a friend that they try self-injection using DMPA-SC?    *(read answer options)* | Very unlikely  Somewhat unlikely  Somewhat likely  Very likely |  | |
| 437 | Do you want to continue with DMPA-SC in the  future? | No  Yes  Uncertain |  | |
| 438 | Are you considering changing to a different method or stopping using family planning  altogether? | No  Yes, thinking about changing methods  Yes, thinking about stopping family planning | If 437=Yes, skip | |
| *Before we finish, I would like to read you two statements about personal confidence. For each, please*  *tell me if you Strongly disagree, Disagree, Agree, or Strongly Agree. Please be honest about how you are feeling today.* | | | | |
| 439 | You feel that you are a person of worth, at least on an equal plane with  others | Strongly disagree  Disagree  Agree  Strongly agree |  | |
| 440 | You feel that you have a number of good qualities | Strongly disagree  Disagree  Agree  Strongly agree |  | |
| 441 | You feel that you do not have much to be proud of | Strongly disagree  Disagree  Agree  Strongly agree |  | |
| 442 | You feel equal to your peers (e.g. sisters, friends, colleagues, etc) | Strongly disagree  Disagree  Agree  Strongly agree |  | |
| ***Those are all the questions I have for you today.***    **IF THIS IS THE 3 OR 6 MONTH FOLLOW-UP, PLEASE READ: *Remember that a study team member will follow-up with you after your next injection using the preferred method (telephone or home visit)***    **IF THIS IS THE 9 MONTH FOLLOW-UP, PLEASE READ: *Thank you for participating in this study. This is our final interview*** | | | | |
| 5. **DISCONTINUATION INTERVIEW** ONLY COMPLETE THIS SECTION ONLY IF 301=No or 401=No | | | | |
| *501* | ***DATA COLLECTOR***  *Record the date of the injection that the woman*  *missed* | *(DD/MM/YY) / /* |  | |
| 502 | Why have you not had an injection since the last interview? | Forgot  Decided to stop using  Unable to visit clinic  No longer had subsequent injections |  | |
| *There is no penalty for changing your mind about using the injectable or about using family planning. I’d like to better understand your reasons for stopping the injectable method you had selected when we first spoke.* | | | | |
| 503 | Why have you decided not to continue using the injectable method?    *(Do not read responses. Record all mentioned)* | Became pregnant or suspect pregnancy  Want to become pregnant  Infrequent sex  No longer fertile  Concerned about health or side effects  Did not trust the product to work  Negative rumors about product  Husband is opposed  Other family members are opposed  Advised not to use product  Preferred to use a different method  Not convenient  Difficult to return to clinic (IM users)  Difficult to self-inject (SC users)  Other family member borrowed the units  I lost my given doses (units)  I did not receive the unit at all to self-inject  Other (specify) |  | |
| 504 | Did you have specific concerns about health problems or side effects? | No  Yes |  | |
| 505 | What were the specific side effects or health problems that were of concern to you? | *Open text* | *If 504=No, skip* | |
| 506 | Did your provider ever discuss your concerns with  you? | No  Yes  Don’t remember |  | |
| 507 | Are you or your husband currently using a different  family planning method? | No  Yes |  | |
| 508 | What method are you or your husband using? | Female sterilization  Male sterilization  Daily pill  IUD/coil  Depo injectable  DMPA-SC injectable  Implant  Male condoms  Female condoms  Emergency contraception  Lactational amen. method  Rhythm/Moon beads/Safe days  Withdrawal  Other(specify) | *If 507=No, skip* | |
| 509 | Why did you switch to DMPA-SC from IM?    **Do not read responses. Record all mentioned** | Fewer side effects  Less painful  Available from LHW/clinic  More widely available, generally  Negative rumors about IM method  Recommended by others  More effective  More safe  Needle size  Other (specify)  Don’t know | *Only ask if IM user and 508=6* | |
| 510 | How many weeks ago did you start using the new  method? | < 1 week ago  1-2 weeks ago  >2 weeks ago | *If 507=No, skip* | |
| 511 | Are you or your husband planning to start using a  family planning method? | No  Yes  Don’t know | *If 507=Yes, skip* | |
| 512 | What method do you plan to use? | Female sterilization  Male sterilization  Daily pill  IUD/coil  Depo injectable  DMPA-SC injectable  Implant | *If 511= No or don’t*  *know, skip* | |
| 513 | When do you plan to start using family planning again? | In the next month  In the next 1 to 6 months  In the next 6 months to 1 year  In 1 to 2 years  > 2 years  Don’t know | *If 511= No or don’t*  *know, skip* | |
| 514 | In general, how satisfied were you with the injectable method? | Very dissatisfied  Slightly dissatisfied  Slightly satisfied  Very satisfied |  | |
| 515 | Why were you satisfied with the injectable  method? | *Open text* | *If 514=1 or 2, skip* | |
| 516 | Where were you not satisfied with the  injectable method? | *Open text* | *If 514 = 3 or 4, skip* | |
| 517 | How likely are you to recommend to a friend that they try this injectable method?    *(read answer options)* | Very unlikely  Somewhat unlikely  Somewhat likely  Very likely |  | |
| 518 | Do you want to have another child? | No  Yes  Uncertain |  | |
| 519 | How soon would you like to have another child? | In the next year  Between 1 and 2 years from now  Between 2 and 5 years from now  Greater than 5 years from now  Don’t know | *If 518= No or Uncertain, skip* | |
| *Before we finish, I would like to read you two statements about personal confidence. For each, please tell me if you Strongly disagree, Disagree, Agree, or Strongly Agree. Please be honest about how you are*  *feeling today.* | | | | |
| 520 | You feel that you are a person of worth, at least on an equal plane with others. | Strongly disagree  Disagree  Agree  Strongly agree |  | |
| 521 | You feel that you have a number of good qualities | Strongly disagree  Disagree  Agree  Strongly agree |  | |
| 522 | You feel that you do not have much to be proud of | Strongly disagree  Disagree  Agree  Strongly agree |  | |
| 523 | You feel equal to your peers (e.g. sisters, friends, colleagues, etc) | Strongly disagree  Disagree  Agree  Strongly agree |  | |
| **That is all the questions I have for you. Your participation in the study is over. Thank you for taking**  **the time to help us with this research.** | | | | |
| ***END INTERVIEW.*** | | | | |

**Tool C – Home observation checklist for DMPA-SC self-injection**

| **Observation Information** | | | |
| --- | --- | --- | --- |
| 101 | Observer Name |  |  |
| 102 | Date of Observation | (DD/MM/YY) / / |  |
| 103 | Participant ID Number |  |  |

**This checklist is used to evaluate DMPA-SC Self Injection practice as per the Standard Operating Procedure for Self-Injection. The respondent has received training and a handout to practice the technique at home. The injection steps can be practiced on a salt filled condom. The study participant should demonstrate the practice once in front of the interviewer.**

**READ:**

*Hello I am*____________*from Jhpiego. Thank you for allowing me to visit you today. I am here because we are interested in learning from your experience in using DMPA-SC. I will ask to see where you store your DMPA-SC doses, how you do injections on a model, and where you dispose of waste after the injection is complete. Anything you show or tell me today will remain confidential. At any point if you want to take a break or want me to leave, please let me know. You have a right to stop the visit at any point. However, knowing your experience is important to us. May we get started?*

Yes

No

# **READ**: *Please show me where you store your DMPA-SC doses in your home.*

| **Observing Storage Practices**  Tick “yes or no” based on observation for each item below.. | | **Observation** | |
| --- | --- | --- | --- |
|  |  | **Yes** | **No** |
| ***Storage Standards*** | | | |
| 201 | Was the device stored at room temperature (outside of a refrigerator) |  |  |
| 202 | Storage location was 30 c or below at the time of observation |  |  |
| 203 | Was the device stored out of direct sunlight and heat? |  |  |
| 204 | Was the device stored out of reach of children and animals? |  |  |
| ***Additional Information about Storage*** | | | |
| 205 | Was the device stored in a clay pot? |  |  |
| 206 | Was the device stored in a cupboard? |  |  |
| 207 | Was the device stored on a high shelf? |  |  |
| 208 | Was the device stored in a high drawer? |  |  |
| 209 | If none of the above, where was the device stored? |  | |
| 210 | Why did you store the unit at the place that you just explain? Please share your reasoning? | *(Note down her response)* | |

GIVE HER A MODEL FILLED WITH SALT. ASK HER (OR ANY RELATIVE WHO DOES INJECTION FOR HER) TO DEMONSTRATE STEP BY STEP FROM THE BEGINNING.

ASK HER TO USE THE SELF-INJECTION **HANDOUT** AS A GUIDE WHILE SHE IS PRACTICING SELF – INJECTION (PROVIDE A NEW COPY IF HERS IS NOT AVAILABLE).

# **READ:** *Just as you did in training, please show me all the steps you would take to self-inject DMPA-SC on the model.*

DO NOT INTERRUPT/ CORRECT HER WHILE SHE IS DEMONSTRATING.

| **Injection steps**  Based on observation of her practice, write S (satisfactory), NS (Not satisfactory) or ND (not done) in the box corresponding to each step. | | **Observation** | | |
| --- | --- | --- | --- | --- |
|  |  | **S** | **NS** | **ND** |
| 301 | Step 1: Prepares supplies (device, waste container, job aid/instructions, calendar) |  |  |  |
| 302 | Step 2: Washes hands |  |  |  |
| **303** | **Step 3: Chooses an injection site and cleans it if visibly dirty (with soap and**  **water)** |  |  |  |
| 304 | Step 4: Checks the expiration date on DMPA pouch, |  |  |  |
| 305 | Step 5: Opens pouch by tearing the notch and remove the device |  |  |  |
| 306 | Step 6: Holds the device by the port while mixing. |  |  |  |
| **307** | **Step 7: Mixes the solution by shaking the device for about 30 seconds,** |  |  |  |
| 308 | Step 8: Checks to make sure the liquid is mixed and there is no damage to the device. |  |  |  |
| 309 | Step 9: Holds the device with the needle pointing upward during activation. |  |  |  |
| 310 | Step 10: Holds the device by the port while activating. |  |  |  |
| **311** | **Step 11: Pushes the needle cap and port together to close the gap, to activate the device.** |  |  |  |
| **312** | **Step 12: Removes needle cap** |  |  |  |
| **313** | **Step 13: Gently pinches the skin to form a “tent”** |  |  |  |
| 314 | Step 14: Holds the port of the device while inserting the needle. |  |  |  |
| 314 | Step 15: Inserts the needle into the tent of skin between the thumb and forefinger. |  |  |  |
| 316 | Step 16: **Inserts needle at a downward angle** |  |  |  |
| 317 | Step 17: Inserts the needle completely so that the port is in full contact with the skin. |  |  |  |
| 318 | Step 18: Moves fingers from the port to the reservoir “bubble” while still  pinching the skin. |  |  |  |
| **319** | **Step 19: Squeeze the reservoir slowly to inject for about 5-7 seconds** |  |  |  |
| 320 | Step 20: Removes the needle from the injection site, then let goes of the skin |  |  |  |
| 321 | Step 21: Does not rub the injection site. |  |  |  |
| 322 | Step 22: Discards the device in a puncture-proof sharps disposal container  without replacing the needle cap. |  |  |  |

ASK HER TO SHOW YOU WHERE SHE DOES/WOULD DISPOSE OF THE DEVICE. IF THERE IS A CONTAINER IN WHICH SHE DISPOSES OF THE DEVICE, TAKE NOTE OF WHAT IT IS AND WHERE IT IS KEPT

| **Observing Waste Management Practices**  *Observe/ ask and tick the relevant options “yes or no” as per below item..* | | **Yes** | **No** | **NA** |
| --- | --- | --- | --- | --- |
| 401 | Is the used device stored in a container until it can be safely disposed of? |  |  |  |
| 402 | Is the Container closed with properly fitted cap, or otherwise sealed and  puncture proof? |  |  |  |
| 403 | Is the used unit disposed of in routine waste bins |  |  |  |
| 404 | Is the used unit kept out of the reach of children |  |  |  |
| 405 | Is the used unit returned to community healthcare worker? |  |  |  |

ASK HER TO SHOW YOU HOW SHE’D CALCULATE THE NEXT INJECTION IF HER INJECTION WAS TODAY. REMIND HER THIS IS HYPOTHETICAL AND SHE DOES NOT NEED TO ADJUST HER NEXT DATE FOR INJECTION, YOU ARE JUST HERE TO LEARN HOW SHE DOES THE REINJECTION SCHEDULE. SILENTLY CALCULATE THE REINJECTION DATE YOURSELF SO THAT YOU CAN ANSWER QUESTIONS ON HER ACCURACY.

| **Observing Calculation of Next Injection Date**  *(Observe/ ask and tick the relevant options “yes or no” as per below items).* | | **Yes** | **No** | **NA** |
| --- | --- | --- | --- | --- |
| 501 | Does she calculate the correct reinjection date? |  |  |  |
| 502 | Does she use a calendar to help her calculate? |  |  |  |
| 503 | Does she use another system (other than a calendar) to help her calculate? |  |  |  |
| 504 | Does she make note (write/type) of the reinjection date somewhere? |  |  |  |

**END OF OBSERVATION.** THANK THE WOMAN FOR HER HOSPITALITY AND STUDY PARTICIPATION. OFFER HER THE SMALL TOKEN OF APPRECIATION FOR SHARING HER TIME AND EXPERIENCES.
